# Single-nucleus multiome analysis of human cerebellum in Alzheimer’s disease-related dementia

**DOI:** 10.21203/rs.3.rs-4871032/v1

**Published:** 2024-08-16

**Authors:** Feixiong Cheng, Yayan Feng, Margaret Flanagan, Borna Bonakdarpour, Pouya Jamshidi, Rudolph Castellani, Qinwen Mao, Xiaona Chu, Hongyu Gao, Yunlong Liu, Jielin Xu, Yuan Hou, William Martin, Peter Nelson, James Leverenz, Andrew Pieper, Jeffrey Cummings

## Abstract

Although human cerebellum is known to be neuropathologically impaired in Alzheimer’s disease (AD) and AD-related dementias (ADRD), the cell type-specific transcriptional and epigenomic changes that contribute to this pathology are not well understood. Here, we report single-nucleus multiome (snRNA-seq and snATAC-seq) analysis of 103,861 nuclei isolated from cerebellum from 9 human cases of AD/ADRD and 8 controls, and with frontal cortex of 6 AD donors for additional comparison. Using peak-to-gene linkage analysis, we identified 431,834 significant linkages between gene expression and cell subtype-specific chromatin accessibility regions enriched for candidate cis-regulatory elements (cCREs). These cCREs were associated with AD/ADRD-specific transcriptomic changes and disease-related gene regulatory networks, especially for RAR Related Orphan Receptor A (RORA) and E74 Like ETS Transcription Factor 1 (ELF1) in cerebellar Purkinje cells and granule cells, respectively. Trajectory analysis of granule cell populations further identified disease-relevant transcription factors, such as RORA, and their regulatory targets. Finally, we prioritized two likely causal genes, including Seizure Related 6 Homolog Like 2 (SEZ6L2) in Purkinje cells and KAT8 Regulatory NSL Complex Subunit 1 (KANSL1) in granule cells, through integrative analysis of cCREs derived from snATAC-seq, genome-wide AD/ADRD loci, and Hi-C looping data. This first cell subtype-specific regulatory landscape in the human cerebellum identified here offer novel genomic and epigenomic insights into the neuropathology and pathobiology of AD/ADRD and other neurological disorders if broadly applied.

**Additional Declarations: Yes** there is potential Competing Interest. Dr. Cummings has provided consultation to AB Science, Acadia, Alkahest, AlphaCognition, ALZPathFinder, Annovis, AriBio, Artery, Avanir, Biogen, Biosplice, Cassava, Cerevel, Clinilabs, Cortexyme, Diadem, EIP Pharma, Eisai, GatehouseBio, GemVax, Genentech, Green Valley, Grifols, Janssen, Karuna, Lexeo, Lilly, Lundbeck, LSP, Merck, NervGen, Novo Nordisk, Oligomerix, Ono, Otsuka, PharmacotrophiX, PRODEO, Prothena, ReMYND, Renew, Resverlogix, Roche, Signant Health, Suven, Unlearn AI, Vaxxinity, VigilNeuro pharmaceutical, assessment, and investment companies. Dr. Leverenz has received consulting fees from consulting fees from Vaxxinity, grant support from GE Healthcare and serves on a Data Safety Monitoring Board for Eisai. The other authors have declared no competing interests.

### Background

The cerebellum has traditionally received the most attention for its role in motor coordination.[1] Recently, however, there has been increasing interest in nonmotor functions of the cerebellum, particularly in Alzheimer's disease (AD) and AD-related dementias (ADRD).[2] For example, neuroimaging studies have revealed marked cerebellar atrophy in AD, Parkinson's disease (PD), and frontotemporal dementia (FTD)[3] associated with selective degeneration of intrinsic connectivity networks.[4] Furthermore, subjects with Lewy body disease (LBD) and AD also show significant cerebellar volume loss compared to controls,[5] and both familial and sporadic AD subjects show significant cerebellar Purkinje cell loss and astrocytosis.[6] Subjects with sporadic AD also accumulate amyloid plaques in cerebellar Purkinje and granule cells.[7] Taken together, there is abundant evidence for significant cerebellar neurodegeneration in AD/ADRD.[8,9] However, the pathophysiologic contribution of specific cerebellar cell types in AD/ADRD has not been identified.

To date, large scale genome-wide association studies (GWAS) have revealed ~100 loci that are significantly associated with AD/ADRD.[10] However, nearly 90% of these loci are located within non-coding regions of DNA, rendering it difficult to identify the target genes and interpret their cell-type specific molecular functions in disease.[11] Recent studies have shown that variants in non-coding regions can disrupt cell type-specific *cis*-regulatory elements (CREs), which precisely tune the expression of target gene in a single cell type.[12] Thus, identifying causal variants and interpreting their function requires analysis of the gene-regulatory maps controlled by cell type-specific CREs.[13] Recent advances in single-nuclei multiome technology have enabled simultaneous profiling of gene expression and chromatin accessibility from the same nuclei,[14] providing opportunities to interrogate the regulatory underpinnings responsible for disease relevant transcriptomic features in a cell type-specific manner. This sequencing platform has successfully identified regulatory mechanisms responsible for AD-associated transcriptomic changes in human cortical tissues.[15] However, it has not been applied to studying the pathogenetic role of the cerebellum in AD/ADRD.

Whereas most of the current etiologic and therapeutic research on dementia has focused on individual disease syndromes,[15,16] different types of dementia also have shared genetic and molecular pathophysiology.[17,18] This has important implications for therapeutic interventions. Whether and how different forms of dementia interact physiologically at the cellular and molecular level has been understudied, especially in the cerebellum. To address this, we conducted single-nucleus multiome (snRNA-seq and snATAC-seq) profiles for postmortem human cerebellum and frontal cortex tissues with a varying neuropathologic degree of AD/ADRD. Control materials were derived from subjects lacking documentation of cognitive impairment or dementia upon retrospective electronic medical record review by a board certified subspecialized cognitive behavioral neurologist. We investigated cell subtype-specific active cCREs based on chromatin accessibility data and identified unique disease-associated transcriptomic/epigenomic signatures related to AD/ADRD. We further constructed transcription factor (TF)-mediated gene regulatory networks in human AD/ADRD cerebellum and applied integrated trajectory analysis to characterize cerebellar granule cell states at the epigenomic and transcriptomic levels. Finally, we used colocalization and fine-mapping to identify a panel of causal GWAS variants, which we then integrated with gene-regulatory maps and Hi-C chromatin looping data to prioritize cell types, causal variants, and likely causal target genes implicated in AD/ADRD cerebellum.

### Results

#### A single-nucleus multiome map in human cerebellum

We used 10x Genomics Multiome technology to profile gene expression and chromatin accessibility within the same nucleus isolated from human postmortem cerebellum and frontal cortex (**Table S1**). Frozen cerebellum tissues were obtained from subjects with AD/ADRD (n = 9) and age- and sex-matched non-dementia control subjects (Control, n = 8). The 9 AD/ADRD donors were divided into three disease phenotypes: donors with AD neuropathologic change (AD_CBE, n = 3), donors with diffuse Lewy body disease (DLBD, n = 3), and donors with progressive supranuclear palsy or frontotemporal lobar degeneration (PSP/FTD, n = 3). We also sequenced six AD brains from frontal cortex (AD_FC, n = 6) as a comparator group (**Figure 1A**). Study groups were stratified both clinically and neuropathologically using standard diagnostic neuropathologic criteria from the National Institute on Aging and Alzheimer's Association (NIA-AA) for assessment of AD neuropathologic change (i.e., amyloid-β and Tau tangles), Lewy body disease, hippocampal sclerosis of aging, and vascular lesions.[19] Neuropathological assessments for frontotemporal lobar degeneration with Tau pathology (FTLD-Tau) (i.e. progressive supranuclear palsy and corticobasal degeneration) was performed using standard diagnostic criteria. Similarly, TDP-43 proteinopathies (incorporating FTLD-TDP and Limbic predominant age related TDP-43 encephalopathy) were assessed neuropathologically using standard diagnostic criteria. As expected, we detected significant difference in the density of amyloid-β and dentate nucleus between AD/ADRD and control cerebellum (**Figure 1B**), suggesting neuropathological and volume changes in the cerebellum in AD/ADRD.

After removing low quality nuclei and doublets (**Figure S1** and see Methods), we retained a total of 103,861 sequenced nuclei (both snRNA-seq and snATAC-seq) with an average of 4,490 nuclei per donor. This revealed a median of 1,695 genes and 10,852 ATAC fragments per nucleus. We also performed latent semantic indexing (LSI) analysis on the batch-corrected snRNA-seq and snATAC-seq datasets (**Figure S1F** and see Methods). This identified 8 major cell types in both snRNA-seq and snATAC-seq datasets, including granule cells (Gran), oligodendrocytes (OLs), astrocytes (Astro), microglia (Micro), glutamate neurons (Glu), excitatory neurons (Excit), Purkinje cells (Purkinje), and oligodendrocyte progenitor cell (OPCs) (**Figure 1C**). These clusters were annotated based on expression level (**Figure 1D**) and chromatin accessibility scores (**Figure 1E**) using well-known marker genes (**Figure S2** and **Table S2**). These broad cell types were further divided into 19 high-resolution subclusters and cell states. We identified 5 subclusters in granule cells: granule cell subcluster 1 (Gran_1) enriched for marker genes detected in granule cell progenitors (*ZIC1*, *ZIC2*, and *PAX6*), subcluster 2 (*BARHL1* and *FAT2*) and 3 (*PLXNB2* and *FAT2*) enriched for genes associated with migration and proliferation of cerebellar granule cells, and subcluster 4 (*RELN* and *RBFOX3*) and 5 (*GRM4* and *RBFOX3*) enriched for marker genes associated with mature granule cells (**Figures 1D** and **1E**). These high-resolution cellular subcluster profiles were highly reproducible using subsampled data analysis (**Figure S3**).

We next examined cellular composition of each subcluster in the context of brain regions. Apart from common cell types between cerebellum and frontal cortex, such as oligodendrocytes, astrocytes, and microglia, we found that granule cells and Purkinje cells showed regional heterogeneity between cerebellum and frontal cortex (**Figure S4A**). Specifically, we observed elevated abundance of granule cells in cerebellum (n = 80,645,

78.1%) (**Figure 1F**), consistent with previous findings that granule cells represent the most abundant cell type in the cerebellum.[20] Furthermore, Purkinje cells were uniquely identified in human cerebellum as being able to induce dysregulated autophagy and aberrant mitophagy in different neurodegenerative conditions.[21] Thus, cerebellum showed a distinct cytoarchitecture compared with that of the frontal cortex. We also found consistent cell types and comparable proportions spanning most donors in human AD/ADRD cerebellum (**Figures S4B** and **S4C**). Altogether, these single-nucleus multiome observations suggest that unique cell type and molecular changes in cerebellum may initialize disease pathogenesis and promote disease progression of AD/ADRD neuropathology.

**Identification of candidate CREs in AD/ADRD**

Chromatin accessibility across the genome defines *cis*-regulatory elements that dynamically control gene expression via interactions with TFs.[22] Using the high-resolution snATAC subclusters, we identified 706,652 peaks of open chromatin regions (**Figure S5A**), which were enriched for binding motifs of transcriptional regulators of cerebellar neurogenesis, such as *Regulatory Factor X3 (RFX3)* in granule cells and *Activating Transcription Factor 1 (ATF1*) in Purkinje cells (**Figure S5B**). The activity of these two TFs was further supported by footprinting analysis of snATAC-seq peaks (**Figure 2A**). Notably, disruption of *RFX3* causes dysregulation of neurobiological pathways associated with neurodevelopmental disease,[23] and deficiency of *ATF1* leads to extensive apoptosis of postmitotic neurons in the brain of adult mice.[24] These observations align with granule and Purkinje cell pathology, which involve molecular mechanisms of survival and apoptosis in both *in vivo* and *in vitro* models of neurodegenerative conditions.[25,26]

To identify candidate CREs (cCREs) with accessibility correlated to local gene expression, we next leveraged snMultiome data to perform peak-to-gene linkage analysis on both the broad cell type identity and granule cell subpopulations (**Figure 2B**, see Methods). In total, we identified 425,798 and 769,801 peak-to-gene links using the full dataset and granule subclustered datasets, respectively (see Methods). These included 13,401 linked genes and 431,834 linked peaks with a minimum absolute correlation value of 0.2 (**Figure 2C** and **Table S3**). The median distance between the linked peaks and the transcription start site (TSS) of the linked genes was 73,769 bp, and there was an inverse relationship between absolute correlation value and distance to TSS ($R^2$ = -0.26, Spearman correlation $P < 2.2 \times 10^{-16}$). These gene-linked cCREs together made up 7.2% of the human genome (GRCh38) and contained binding motifs of key transcriptional regulators (**Figure S5C**). Of these gene-linked cCREs, 72.2% were located at least 2 Kb away from annotated promoter regions of protein-coding genes, a proportion supported by CRISPR perturbations of enhancer-promoter connections.[27] Notably, the linked genes were enriched for previously identified AD seed genes[28] (Fisher's exact test $P = 0.0033$), including *APOE*, *TGFB1*, *PICALM* and *APP* (**Figure S6** and **Table S3**). Consistent with previous studies,[29] we identified 1,821 highly regulated genes (HRGs) exceeding an inflection point at 150 when ranking genes by the number of significant correlations, with a much larger number of significant peak-to-gene correlations as candidate enhancers (**Figure 2D**). Those HRGs were significantly enriched for ChIP-seq targeted genes in human brains from the Epigenomics Roadmap Project ($q = 1.07 \times 10^{-10}$, **Table S4**), including TFs orchestrating gene regulatory programs in the maturation of cerebellar granule cells, such as *RNA-Binding Fox-1 Homolog 3* (*RBFOX3*), Neurexin 3-Alpha (*NRXN3*), *Cadherin 18* (*CDH18*), *ETF Variant Transcription Factor 1* (*ETV1*), *Glutamate*

*Metabotropic Receptor 4* (*GRM4*) and *Neuronal Differentiation 1* (N*NEUROD1*).[20] We further performed functional enrichment analysis and revealed that those HRGs were significantly enriched in AD/ADRD-related pathways, such as insulin secretion ($q = 7.16 \times 10^{-6}$), GABAergic synapses ($q = 4.0 \times 10^{-5}$), and spinocerebellar ataxia ($q = 4.7 \times 10^{-5}$) (**Table S5**), suggesting involvement of those HRGs in the gene-regulatory programs in human cerebellum.

We next turned to examine cCRE diversity between human cerebellum and frontal cortex using peak-to-gene linkages analysis. Using a more stringent criteria (correlation $r \geq 0.5$), we identified 13,326 and 3,530 linked genes in cerebellum and frontal cortex, respectively (**Table S6**). We found 2,705 *cis*-regulatory linked genes between cerebellum and frontal cortex, including multiple known AD/ADRD causal genes, such as *Apolipoprotein E* (*APOE*), *Bridging Integrator 1* (*BIN1*), *Arichidonate 5-Lipoxyge*nase (*ALOX5*), and *FYN*. Specifically, we unraveled tightly-linked peaks with *BIN1* in both cerebellum and frontal cortex (**Figure 2E**). We further identified 10,621 linked genes specifically in cerebellum, *including Interleukin* 33 (*IL33*), *Presenilin 1* (*PSEN1*), *Phosphatidylinositol Binding Clathrin Assembly Protein* (*PICALM*) and *Beta-Secretase Beta-Site APP Cleaving Enzyme* (*BACE1*) (**Table S6**). We only identified IL33-linked peaks in cerebellum (**Figure 2F**), suggesting that cerebellum may capture a panel of crucial gene sets involved in pathogenesis of AD/ADRD.

Next, we characterized the effect of disease status on cCREs dynamics in human cerebellum. Here, we detected 86,250 differential accessible regions (DARs) between AD/ADRD and control cerebellum (Wilcoxon false discovery rate (FDR) $< 0.05$, $\log_2 FC \geq 0.3$; **Table S7**). Genes linked to those AD/ADRD affected peaks were significantly enriched in multiple key biological pathways (**Table S8**), including metabolic pathways ($q = 7.40 \times 10^{-18}$), amyotrophic lateral sclerosis ($q = 6.04 \times 10^{-16}$), pathways of neurodegeneration ($q = 3.28 \times 10^{-}$

$^{12}$), neurotrophin signaling pathway ($q = 3.50 \times 10^{-10}$), and spinocerebellar ataxia ($q = 2.00 \times 10^{-9}$). Furthermore, peaks that displaying increased accessibility in AD/ADRD cerebellum were enriched for motifs of key TFs, such as *Zinc Finger and BTB Domain Containing 14* (*ZBTB14*) and *Zic Family Member 2* (*ZIC2*) in mature granule cells, and *Zinc Finger Binding Protein 385D* (*ZNF385D*) and *SP4 Transcription Factor* (*SP4*) in Purkinje cells (**Figure S5C**). Altogether, these results support the involvement of differentially cCREs on the regulatory potential of key genes and TFs implicated in human cerebellum with AD/ADRD neuropathology.

**Cell type-specific transcriptomic changes in AD/ADRD cerebellum**

We next inspected cell type-specific transcriptional cerebellum differences impacted by variations in chromatin accessibility between AD/ADRD and control cerebellum. A total of 1,158 differentially expressed genes (DEGs) were identified between AD/ADRD and control cerebellum (**Figure 3A**). Although the majority of DEGs were cell type-specific, 310 were identified across multiple cell types (**Figure 3B**), including key genes or TFs related to pathological pathways involved in neurodegenerative conditions. For example, *EBF Transcription Factor 1* (*EBF1*) showed significant upregulation in both astrocytes and granule cell subpopulations (**Figure 3B**), which may promote expression of target genes involved in neuronal development.[30] We also observed significant downregulation of *Phosphodiesterase 10A* (*PDE10A*) across the five granule cell subpopulations. Loss of *PDE10A* expression is associated with progression and severity in Parkinson’s disease.[31] Furthermore, there was significant overlap between disease-associated DEGs and cell type-specific marker genes, and between cell type-specific DEGs and cCRE-linked genes within that cell type (Fisher's exact test $P < 0.05$, **Figure 3C**), highlighting a critical role of cCREs in disease-associated transcriptomic changes.

As the pathophysiology of different ADRD conditions may overlap with AD at multiple molecular levels,[32] we next examined DEGs across AD, DLBD, and PSP/FTD and identified a shared set of DEGs (**Figure 3D**). Notably, we identified more upregulated genes in astrocytes and granule cell subclusters in donors with AD, whereas donors with DLBD and PSP/FTD had more downregulated genes. We also identified enrichment of 24 related pathways that were commonly dysregulated across different neurodegenerative ADRDs ($q < 0.05$), including cAMP signaling pathway, cGMP-PKG signaling pathway, oxidative phosphorylation, and neurodegeneration pathways (**Figure 3E**). These results suggest shared molecular mechanisms across different ADRDs.

Given the convergent dysregulated pathways across multiple ADRDs, we next turned to identify unique disease-specific gene regulation of cCREs. We focused on DEGs and peaks within 250 Kb from each other. Examination of DEGs-linked peaks identified 81.4% (n = 943) of differential genes between AD/ADRD and control cerebellum had a linked peak in the same cell type. We then examined the overlap trend between DEGs-linked peaks and the identified differentially accessible regions (DARs) in AD/ADRD cerebellum. Of those dysregulated genes, we identified 70.6% (n = 666) DEGs that were linked to at least one differential accessible peak. Those *cis*-regulated DEGs (n = 666) were significantly enriched in pathways that are hallmarks of neuronal and brain functional development, such as Parkinson disease ($q = 6.12 \times 10^{-14}$), pathways of neurodegeneration ($q = 4.43 \times 10^{-10}$), synaptic vesicle cycle ($q = 2.50 \times 10^{-5}$), and mitophagy ($q = 9.08 \times 10^{-3}$) (**Table S9**). Here, we highlighted the most dysregulated genes *Calmodulin 1* (*CALM1*) ($log_2FC = 2.12$, $q = 1.85 \times 10^{-4}$), *Transmembrane*

*Protein 150* (*TMEM160*) ($\log_2$FC = 2.59, $q$ = 0.015) and *Rho GDP Dissociation Inhibitor Gamma* (*ARHGDIG*) ($\log_2$FC = 2.65, $q = 1.35 \times 10^{-7}$) in mature granule cells, Purkinje cells and astrocytes, respectively, which could be related to chromatin accessibility changes between AD/ADRD and control donors (**Figure 3F**): (1) *CALM1* plays an essential role in neuronal migration;[33] (2) *TMEM160* contributes to neuroimmune signaling in injured mice;[34] and (3) *ARHGDIG* is a GDP-dissociation inhibitor for Rho proteins that plays a primary role in modulating the activity of GTPases.[35] These unique epigenetic and transcriptional changes in human cerebellum are thus likely to play crucial roles in the underlying pathobiological pathways of AD/ADRD.

**Gene-regulatory networks in human AD/ADRD cerebellum**

We next sought to identify TFs involved in active cCREs and target genes. To further investigate the regulatory roles of links, we identified peak-gene-TF "trios" in which (1) there was a correlation between the peak and gene, (2) the accessibility of a linked peak harboring a specific TF motif was correlated with the mRNA level of that TF, and (3) the expression of the TF was associated with the expression of the linked genes (**Figure 4A**). We restricted our analysis to links with significant correlations within 250 Kb of the linked gene's transcription start site (TSS). In total, we identified 165 peak-gene-TF trios involving 60,011 unique peaks and 8,787 genes in human cerebellum (**Table S10**). Specifically, 17,050 (10.5%) of the peaks in these trios are observed in promoters, with the majority present in intronic (n = 47,104, 33.6%) and upstream 5 Kb (n = 29,357, 20.9%) regions (**Figure S7**). Trio peaks are significantly enriched for ENCODE distal (Fisher's exact test $P < 2.2 \times 10^{-16}$) and proximal ($P < 2.2 \times 10^{-16}$) enhancer-like sequences, and there is a median of 106 genes and 363 peaks per

TF. We further generated a panel of cell type-specific TF regulatory networks with a larger number of peaks (n ≥ 200) and genes (n ≥ 40) (**Figure 4B** and **Table S10**). For example, downregulated *RORA* was significantly associated with 631 regions and 423 genes in Purkinje cells. Dysregulated *RORA* is likely a driving force for neurodevelopmental disorders by affecting target genes involved in inflammation.[36] In addition, the transcription factor *ELF1* was significantly associated with 375 regions and 44 genes in mature granule cells, and activation of *ELF1* was involved in functional roles of brain cells.[37]

We next used snMultiome data to examine the regulatory role of *RORA* and *ELF1* in Purkinje and mature granule cells, respectively. Motif variability analysis revealed that *RORA* and *ELF1* showed significantly elevated activity in Purkinje (**Figure 4C**) and mature granule cells (**Figure 4D**), respectively. Cell type-specific subtracted activity of *RORA* and *ELF1* was also supported by footprinting analysis of snATAC peaks, which revealed motif centers to be protected from Tn5 transposition, consistent with *RORA* and *ELF1* occupancy (**Figures 4C and 4D**). The activity level of transcription factors can exert its regulatory potential on its target genes, and we observed that the activity of target genes of *RORA* was related to upregulation in Purkinje cells (**Figure 4C**). By contrast, target genes of *ELF1* showed downregulation in mature granule cells (**Figure 4D**). These results indicate that *RORA* acts as a transcriptional activator in cerebellar Purkinje cells and *ELF1* acts as a transcriptional repressor in mature cerebellar granule cells in AD/ADRD, providing molecular insights into how *RORA* and *ELF1* contribute to AD/ADRD pathophysiology.

To gain further insight into TF-mediated gene regulation in AD/ADRD cerebellum, we next constructed cell type-specific TF regulatory networks for *RORA* and *ELF1*. For a given TF, we identified candidate target genes as those whose promoters or linked cCREs are accessible and contain the TF's binding motif in the cell type of interest. We repeated this process for *RORA* and *ELF1*, generating Purkinje-specific (**Figure 4E**) and mature granule-specific (**Figure 4F**) TF-gene regulatory networks. We found that target genes of *RORA* were significantly enriched in pathways related to different neurodegenerative conditions, such as Parkinson disease ($q$ = 0.010) and AD ($q$ = 0.047). This identified key genes located at known AD/ADRD GWAS loci and multiple AD/ADRD DEGs, including *Seizure Related 6 Homolog Like 2* (*SEZ6L2*) ($log_2FC$ = 1.76, $q$ = 0.0056). Elevated *SEZ6L2* has been observed in adult AD brains[38] and contributes to refinement of synaptic connectivity between climbing fibers and Purkinje cells in the cerebellum.[39] We also examined *ELF1*-mediated regulatory networks in mature granule cells and found that target genes of *ELF1* were enriched in gene signatures related to longevity regulating pathways ($q$ = 0.008). In addition to genes located at known AD GWAS loci, we identified multiple dysregulated genes in AD/ADRD cerebellum, such as upregulated *KAT8 Regulatory NSL Complex Subunit 1* (*KANSL1*) ($log_2FC$ = 1.33, $q = 4.32 \times 10^{-7}$). *KANSL1* dysregulation has been reported to promote cell senescence by regulating the ras/mitogen-activated protein kinase (MAPK) pathway.[40]

**Integrated trajectory analysis identifies TFs and genes involved in granule cell differentiation**

Granule cells undergo continuous replacement by coordinated birth, proliferation, differentiation, and migration into mature granule cells.[20] Thus, we examined whether the identified TF-mediated gene regulatory networks were involved in granule cell heterogeneity in human cerebellum. Specifically, we constructed a semi-supervised pseudotemporal trajectory using 80,645 nuclei from our snMultiome data to recapitulate the known maturation process of granule cell progenitors, proliferating granule cells, and mature granule cells (**Figure 5A**). We obtained a similar differentiation process when using the ratio of spliced and unspliced messenger RNA for an individual gene at a given time point (**Figure 5B**). To identify TFs with a regulatory role in specifying granule cell subpopulations, we first identified motifs with variable chromatin accessibility across granule cell maturation. We then correlated TF expression with motif activity across granule cell subsets to differentiate between TFs with similar motifs. Of the 165 TFs identified in TF-gene regulatory networks, we found significant enrichment along the granule cell trajectory analysis ($P = 1.28 \times 10^{-5}$, **Figure S8A**). We showcased two key TFs in granule cell populations: *Transcription Factor 12* (*TCF12*) and *RORA*. We found that *TCF12* was highly expressed in early trajectory granule cell progenitors, while *RORA* was highly expressed in mature granule cells (**Figure 5C**). *TCF12* is essential for neuronal migration in cortical development ,[41] and *RORA* play crucial roles of cerebellar and systemic abnormalities observed in neurodevelopmental disorders.[36]

We next examined gene signatures involved in the granule cell trajectory process. We found elevated chromatin accessibility and gene expression signature of mature granule cells at the end of the trajectory, whereas the granule cell progenitor signatures decreased (**Figure S8B**). Visualization of the most variable 10% of peaks along this trajectory revealed a continuous, gradual opening and closing of accessible chromatin (**Figure 5D**). The most variable 10% of genes included known transcriptional changes during granule cell differentiation, with early trajectory cells expressing granule cell progenitor (*CALM1* and *CALM2*),[33] middle cells expressing proliferating granule cells (*Phospholipase C gamma 2* (*PLCG2*) and *Dipeptidyl Peptidase Like 6* (*DPP6*)) and later cells expressing mature granule cells (*Phosphodiesterase 4B* (*PDE4B*) and *Ring Finger Protein 152* (*RNF152*)).[20] We further found significant enrichment of target genes of the 165 TFs involved in granule cell trajectory (*P* = 0.0032). Specifically, genomic tracks of *PDE4B* and *RNF152*, active in mature granule cells, reveal coordinated changes in gene expression and linked chromatin accessibility across differentiation trajectory (**Figures 5E and 5G**).

**Cell type-specific *cis*-regulation at AD/ADRD genetic loci**

To determine if the cCREs were significantly enriched for GWAS loci associated with complex brain-related disorders, we performed cell type-specific linkage-disequilibrium score regression (LDSC) analysis in our snATAC-seq subclusters using GWAS summary statistics in AD and other relevant neurodegenerative traits, including PD, ALS, PSP, LBD, and cerebellar volume (see Methods, **Table S11**). Mature granule cells showed a significant enrichment for three published AD GWAS studies (**Figure 6A**).[10,42,43] Because GWAS SNPs could alter chromatin accessibility resulting in differences in disease susceptibility between individuals, we further investigated enrichment of GWAS loci in the cell type-specific differentially accessible peaks (**Figure 6B**). We found that AD GWAS loci were significantly enriched in differentially accessible peaks in granule cells (Fisher's exact test *P* = 0.032) and Purkinje cells (*P* = 0.045). Specifically, PD GWAS loci were most strongly enriched in differential accessible peaks in granule cell subpopulations. The results of this GWAS heritability analysis indicate that variants in disease-relevant peaks may have a greater contribution to disease susceptibility of AD/ADRD.

After nominating disease-relevant cell types in AD/ADRD cerebellum, we sought to identify likely causal genes associated with GWAS loci. We first complied a comprehensive catalog of putative disease-relevant SNPs in AD and other relevant traits, including PD, ALS,

PSP, LBD, and cerebellar volume (see Methods, **Table S11**), considering the propensity of nearby SNPs to be co-inherited based on linkage disequilibrium (LD). We identified (1) any SNPs passing genome-wide significance ($P = 5.0 \times 10^{-8}$) in GWAS summary statistics data, (2) any SNPs exhibiting colocalization of GWAS and cerebellum expression quantitative trait loci signal (coloc/FINEMAP colocalization posterior probability $> 0.01$) and (3) any SNPs in LD with a SNP in the previous two categories based on an LD $R^2$ value $\geq 0.8$ calculated from the 1000 Genomes dataset. We identified 3,884 SNPs across 196 loci associated with AD, 4,579 SNPs across 160 loci associated with PD, 656 SNPs across 144 loci associated with ALS, 662 SNPs across 65 loci associated with cerebellar volume, and 141 SNPs across 29 loci associated with LBD (**Table S12**). By overlapping the co-accessibility maps with chromatin accessibility signal and GWAS statistics along the genomic axis, we unraveled 29 AD fine-mapped GWAS loci linked genes, the majority of which were differentially expressed in a cell type-specific manner (**Figure 6C** and **Table S13**). These genes included previously nominated AD GWAS genes, including Microtubule Associated Protein 2 (*MAPT*), Speedy/RINGO Cell Cycle Regulator Family Member E3 (*SPDYE3*), Nuclear Receptor Subfamily 1 Group H Member 3 (*NR1H3*), and Proteasome 26S subunit ATPase 3 (*PSMC3*) and immune modulators of TNF-alpha signaling pathway (Mitogen-Activated Protein Kinase Kinase Kinase 3 [*MAP3K3*] and MAP Kinase Activating Death Domain [*MADD*]). We further identified 12 PD fine-mapped GWAS loci linked genes, such as genes involved in chromatin organization (*KANSL1* and SET Domain Containing 1A Histone Lysine Methyltransferase [*SETD1A]*). Furthermore, we also identified Beta-1,4-galactosyltransferase 5 (*B4GALT5*) and Golgi Brefeldin A Resistant Guanine Nucleotide Exchange Factor a (*GBF1*) as harboring the potential *cis*-regulatory relationships disrupted by likely causal variants in ALS and cerebellar volume GWAS loci (**Figure 6C** and **Table S13**).

We next compared transcriptomic changes of those fine-mapped GWAS linked genes between AD and ADRD cerebellum and observed concordant expression changes between AD and ADRD cerebellum (**Figure 6C**). Using *SEZ6L2* as an example, we found one linked fine-mapped SNP rs4788201 (GWAS $P = 2.04 \times 10^{-9}$) and 283 linked peaks. *SEZ6L2* showed significant upregulation in AD/ADRD cerebellum in Purkinje cells ($\log_2$FC = 1.76, $q = 0.0056$), with elevated expression that were more significant in ADRD cerebellum ($\log_2$FC = 3.88, $q = 1.31 \times 10^{-11}$). We further detected frequent linked peaks (n = 373) and 12 fine-mapped SNPs with *KANSL1*, as well as significant upregulation of *KANSL1* in AD ($\log_2$FC = 0.69, $q = 0.0017$), ADRD ($\log_2$FC = 1.47, $q = 4.95 \times 10^{-5}$) and AD/ADRD ($\log_2$FC = 1.33, $q = 4.32 \times 10^{-7}$) cerebellum in mature granule cells. These observations illustrate a shared molecular catalog across AD and ADRD (such as DLB and PSP/FTD) in human cerebellum (**Figure 6**).

**Identifying likely causal genes** (*SEZ6L2 and KANSL1*) **in human AD/ADRD cerebellum**

After nominating cell types and target genes associated with AD/ADRD cerebellum, we sought to identify causal relationships by identifying SNPs that might directly interact with promoters of target genes. Human cerebellum Hi-C data showed that chromatin looping occurs between putative regulatory regions containing fine-mapped SNPs and the promoters of 9 genes (**Figure S9**), including *CELF1*, *KANSL1*, *KCTD13*, *PPP4C*, *SEZ6L2*, *STAG3*, *TMEM219*, *YPEL3* and *B4GALT5*. For the remaining loci, multiple SNPs were found in the introns of target genes and/or intergenic regions but lacked strong chromatin-looping-mediated SNP-gene interactions. These SNPs may act through other mechanisms such as perturbation of pre-messenger RNA splicing or altered noncoding RNA stability, structure and/or function.[44]

One high-effect GWAS SNP is rs4788201 (GWAS $P = 2.04 \times 10^{-9}$) in upstream of *SEZ6L2* from the published GWAS study by Bellenguez et al.[10] (**Figure 7A**). The alternative allele of rs4788201 was associated with significant increase of chromatin accessibility in AD/ADRD cerebellum in Purkinje cells from our scATAC-seq data analysis (chr16:29961225-29961725, $\log_2$FC = 0.74). Using Hi-C chromatin looping data, we detected frequent interactions from this peak and promoters of *SEZ6L2*. The regulatory potential of rs4788201 was further confirmed by the significant transcriptomic changes of *SEZ6L2* in cerebellum Purkinje cells ($\log_2$FC = 1.76, $q = 0.0056$). *SEZ6L2* is physiological substrate of *BACE1* in neurons and is overexpressed in Purkinje cells.[45]

We also identified another genome-wide significant SNP rs62056801, which resides in the intron region and is linked to *KANSL1* expression (**Figure 7B**). This SNP showed genome-wide significance for AD GWAS SNPs by Bellenguez et al.[10] (GWAS $P = 2.71 \times 10^{-8}$) and PD GWAS SNPs from the study by Nalls et al.[46] ($P = 1.46 \times 10^{-20}$). Our scATAC-seq data suggested that the alternative allele of rs62056801 was significantly associated with elevated chromatin accessibility in AD/ADRD cerebellum in granule cell subpopulation 4 (chr17:45902079-45902579, $\log_2$FC = 0.32). We identified frequent Hi-C chromatin looping between this putative regulatory region and promoter of target gene *KANSL1*, suggesting a mechanistic pathway for this causal variant. Furthermore, this SNP has been identified as a significant eQTL for *KANSL1* expression in human cerebellum (eQTL $P = 6.49 \times 10^{-10}$). The regulatory capability of rs62056801 was further confirmed by the significant upregulation of *KANSL1* in granule cell subpopulation 4 ($\log_2$FC = 1.33, $q = 4.32 \times 10^{-7}$). Dysregulated

*KANSL1* resulted in imbalanced autophagy and synaptic deficits in human brains.[47] In summary, these integrative genetics and snMultiome data analysis identifies likely causal genes with potential biological pathways involved in AD or multiple ADRD types. Further experimental validations are highly warranted to validate causal relationships and elucidate functional roles of variants/genes (**Figure 7**) with multiple ADRD pathogeneses in the future.

### Discussion

We presented a comprehensive epigenomic and transcriptomic atlas of human AD/ADRD cerebellum using a single-nucleus multiome analytic approach. This enabled molecular investigation of underlying neuropathological changes within human AD/ADRD cerebellum, which was previously described only by neuroimaging and anatomical studies.[2,4] We also identified unique transcriptomic and epigenomic signatures and gene networks/pathways across diverse cell types or subpopulations, as well as how molecular interactions between TF binding sites and cCREs contribute to those transcriptomic changes. We used these gene regulatory networks to prioritize causal variants, cell types and target genes implicated in the pathobiology of cerebellum-related neurodegenerative conditions. Thus, our single-nuclei multiome analysis supports molecular underpinnings of the potential pathological involvement of the cerebellum in AD/ADRD.

This study generated high-quality single-nucleus multiome profile for AD/ADRD cerebellum. The resulting chromatin accessibility profiles clustered by cell types shared significantly higher proportion with those from a public human aging cerebellum snATAC-seq dataset (**Figure S10**). Specifically, we observed that these snATAC peaks were concordant among donors and included more than 95% of peaks from a published snATAC-seq study of human aging cerebellum (**Figure S10**).[48] This indicates that our snMultiome data can recapitulate open chromatin regions from normal aging human cerebellum.

We also found distinct regional heterogeneity of cell composition between cerebellum and frontal cortex, especially for the significant increase of granule and Purkinje cells in cerebellum (**Figure 1F**). This significant expansion of granule cells plays a crucial role in cerebellum neuronal networks and development.[49] It has been demonstrated that cultured granule cells from rat cerebellum can upregulate molecules of both apoptosis receptor-mediated and mitochondrial-mediated death pathways.[50] Conversely, gene expression alterations in granule cells of transgenic mice are associated with synaptic changes and inflammatory response. As the solo output of the cerebellar cortex, Purkinje cells are among the most highly vulnerable population of neurons to programmed cell death in response to intrinsic diseases or extrinsic signals.[25] Indeed, researchers have observed early loss of cerebellar Purkinje cells in human and mouse AD brains.[51] This cellular alteration can induce pathologic changes in neurofilament phosphorylation states within Purkinje cells in human cerebellum with multiple sclerosis.[52] Purkinje cell degeneration has also been shown to yield activated autophagy and enhanced mitophagy in mice.[21] Thus, the observed cytoarchitecture changes in cerebellum may reflect key cell populations that are vulnerable to neurodegenerative conditions.[53]

Another contribution of this study is that we profiled cell type-specific and disease-specific *cis*-regulatory landscapes in human AD/ADRD cerebellum. These may mediate gene expression changes in late-stage AD/ADRD cerebellum, along with TFs whose activity are predictive of target gene expression. Joint analyses of the transcriptome and chromatin profiles in the same nuclei enables greater confidence in combining the correlations between cCREs and TF binding motifs and between TF activity and target gene expression.[15] Through peak-to-gene linkage analysis, we found common and unique transcriptomic and epigenomic signatures and associated gene networks in AD cerebellum with frontal cortex, such as the key AD GWAS-linked gene *BIN1*[54] and *IL33*[55], providing potential targets for further study in AD cerebellum. Furthermore, we identified significant negative correlations between *RORA* activity and its target gene expression in Purkinje cells, suggesting a gene-silencing role of *RORA* for selected targets. As a key TF in cerebellar development, dysregulation of *RORA* in Purkinje cells reduces expression of genes controlled by *RORA*, which determines disease severity in adult spinocerebellar ataxia type 1 (SCA1) mice.[56] We also identified motif activity of *ELF1* as having significant positive correlation with expression of its gene targets in mature granule cells. Gene targets of *ELF1* are enriched in longevity regulating pathway.[57]

Using this regulatory landscape, we profiled cell type-specific gene expression changes of human cerebellum across different neurodegenerative conditions. We found that both diagnosis DEGs and cell type-specific DEGs exhibited significant overlap with cCRE-linked gene sets, especially for upregulated $Ca^{2+}$ sensors *CALM1* and *TMEM160* in granule and Purkinje cells, respectively. *CALM1* is related to tau phosphorylation and neuronal function[58,59] and *TMEM160*, localized in the inner mitochondrial membrane, may suppress reactive oxygen species (ROS) generation and stabilize mitochondrial proteins.[60] Further study of the candidate regulatory elements we identified for those DEGs could improve our understanding of how its gene targets become dysregulated in AD/ADRD cerebellum and affect disease progression. We further found shared pathological pathways between AD and ADRD cerebellum, especially in granule and Purkinje cells. While AD is the most common dementia diagnosis, ADRDs share many cognitive and pathological features with AD and can be difficult to distinguish from

AD.[61] Combining evidence from human genetic studies, these converging genotype-phenotype interactions can provide further insight into these conditions.[62,63]

Using LDSC and fine-mapped SNP enrichment, our analysis also revealed driver cell types and disease-specific patterns of genes implicated in inherited AD/ADRD risk by GWAS loci. We further prioritized two candidate therapeutic targets using Hi-C data derived from human cerebellum, including *SEZ6L2* in Purkinje cells and *KANSL1* in mature granule cells. *SEZ6L2* is a brain-specific receptor-like protein, whose overexpression can predict poor prognosis in cancer patients.[64] We found a potential causal SNP rs62056801 for both AD and PD GWAS loci, which may affect *KANSL1* expression by increasing the cCRE chromatin accessibility in AD/ADRD cerebellum. *KANSL1* plays a crucial role in regulating mitophagy and is a novel gene for drug targeting in PD.[65] However, we were unable to identify a potential causal SNP for many GWAS loci across different neurodegenerative conditions, perhaps because the SNPs were not identified among the shared genetic etiology of multiple neurodegenerative disorders,[66] and the observed disease-related peaks were observed only in PD and AD shared genetic loci. Our results lay the groundwork for mechanistic studies in appropriate cellular context to confirm the deleterious nature of noncoding GWAS SNPs prioritized in this study.

We acknowledged several potential limitations. One limitation is that our current sample size is small although we have enough statistical power during single-nuclei multiome sequencing data. Second, our data has a relatively low number of cells profiled from rare cell types like microglia, which can exhibit a dynamic, transcriptional, and immunological profile within AD/ADRD cerebellum.[67,68] Expansion of the current studies with more cells and donors would help resolve more disease-relevant rare cell subtypes and reveal cell type-specific peaks of chromatin accessibility that may have been missed in the current analyses using single-cell foundation models. Finally, while our findings provide a valuable framework for linking genetic variation to disease phenotypes, the SNP-to-gene regulatory interactions and the underlying regulatory elements should be further validated in appropriate cellular contexts in future studies.

### Methods

#### Human brain tissues

Postmortem human brain biospecimens were obtained from the Northwestern University that were collected for the brain biobank from the general hospital autopsy service with appropriate consent obtained for research use. As all samples for this study were from this source, and no clinical information was obtained during life from the decedents, samples were exempt from human subjects' research requirements. Post-mortem biospecimens that are appropriately deidentified are exempt from requiring an IRB protocol, specifically. Clinical group determination was performed via retrospective electronic medical record review by a board certified subspecialized cognitive behavioral neurologist for all groups. Frozen brain tissues were collected from the following groups of subjects: tissues from the frontal cortex of donors retrospectively clinically classified as having Alzheimer's disease (n = 6), tissues from the cerebellum of donors retrospectively clinically classified as having Alzheimer's disease and Alzheimer's disease related dementia (AD/ADRD, n = 9), and tissues from unaffected cognitively healthy cerebellum (n = 6). The nine AD/ADRD patients contained three disease types, including Alzheimer's disease neuropathologic change (AD, n = 3), diffuse Lewy body disease (DLBD, n = 3), Progressive Supranuclear Palsy (PSP, n = 3). AD/ADRD donors were clinically diagnosed based on National Institute on Aging and Alzheimer's Association criteria.[19] All diseased subjects had a clinical diagnosis of neuropathological changes and evidence of amyloid-β and dentate nucleus. The collected samples were stored in 1 × PBS at 4 °C before downstream processing. Metadata information for each subject is presented in **Table S1**.

**Nuclei isolation from human brain tissues**

Approximately 20-30 mg of frozen human brain tissue per sample were transferred into a sterilized 2 mL dounce homogenizer. Before the tissues were thawed, we added 2 mL chilled NP40 lysis buffer (10 mM Tris-HCl, pH 7.5, 10 mM NaCl, 3 mM $MgCl_2$, 0.1% Nonidet P40 Substitute, 1 mM DTT, 1 U/μL RNase inhibitor) into the dounce homogenizer. The tissues were further homogenized 15× using A Pestle, and 10× using B pestle before transferred into a centrifuge tube to incubate 2 min on ice. We further added 2 mL wash buffer containing PBS, 1% BSA and 1 U/μL RNase inhibitor into the tube. The lysed tissue was centrifuged at >500 × g for 5 min at 4°C. Two more times of washing was followed with wash buffer and filtered through 40 μm cell strainer. After centrifugation, the supernatant was removed by aspiration and the nuclear pellet was resuspended in 0.5 mL wash buffer with 5 uL 7-AAD solution and BD FACSAria™ Fusion cell sorter.

**snMultiome library generation, sequencing**

Paired Single cell ATAC + RNA-seq libraries were prepared using the Chromium Next GEM Single Cell Multiome ATAC + Gene Expression platform (10X Genomics, Pleasanton, CA). Briefly, human brain nuclei were isolated, washed, and counted for use with single nuclei multiomics analysis according to the manufacturer's instructions (10x Genomics, CG000338 Rev E) with modified/optimized for our samples. Nuclei suspension was first incubated in a transposition mix. Thereafter, along with the single cell multiome gel beads and partition oil, the single nuclei master mixture containing tagmented single nuclei suspension was transferred onto a Next GEM Chip J, and the chip was loaded to the Chromium Controller for GEM generation and barcoding. Barcoded transposed DNA and cDNA were amplified after GEMs being released. At each step, the quality of cDNA, ATAC library and cDNA library was examined by Bioanalyzer. The final single indexed ATAC libraries and the dual indexed gene expression libraries were sequenced on an Illumina Novaseq 6000, with index reads of 10 bp + 24 bp, and 100 bp paired-end reads.

**snMultiome quality control, dimensionality reduction and cluster analysis**

Initial processing of snMultiome fastq data was first aligned to the GRCh38 (refdata-cellranger-arc-GRCh38-2020-A-2.0.0, 10x Genomics) reference genome and quantified using 'cellranger-arc count' algorithm according to Cell Ranger ARC algorithms overview (https://www.10xgenomics.com/support, v 2.0.2). We next used ArchR functions to process the filtered peak-barcoded and gene-barcoded matrix.[69] We then used 'createArrowFiles' function to filter low-quality nuclei based on chromatin accessibility data (TSS enrichment ≥ 3 and nFragment ≥ 1000). We then computed doublet scores using 'addDoubletScores' function and samples with projection score $R^2 > 0.9$ were used for doublet inference. Inferenced doubles were filtered using 'filterDoublets' function in ArchR. We then performed dimensionality reduction by using Iterative Latent Semantic Indexing (LSI) that has been depth normalized to a constant (10,000) followed by normalization with the inverse document frequency and then log-transforming the resultant matrix. The final clusters were determined using the 'addClusters' function with a resolution of 0.4 and max clusters of 35. This clustering procedure generated a two-dimensional representation of the data using the 'addUMAP' function with nNeighbors = 50 and minDist = 0.4. After removal of poor-quality clusters composing nuclei from a single sample (clusters 3, 6, 7, 14, 19), we obtained 19 final clusters for subsequent analysis. We used the 'getMarkerFeatures' function in ArchR to identify marker genes based on gene activity score and gene expression data. We then assigned each cluster to putative cell types and states based on known marker genes in human and mouse cerebellum (**Table S2**). In the granule cell we identified three different cell states and five subclusters, characterized by high gene expression and gene score for granule cell progenitor (*ZIC1*, *ZIC2*, and *PAX6*), proliferating granule cells (*BARHL1*, *FAT2*, and *PLXNB2*) and mature granule cells (*RELN*, *GRM4* and *RBFOX3*). We computed proportions of cells from AD/ADRD or control samples. A two-tailed Wilcoxon rank-sum test was used to check cell proportion changes between AD/ADRD and control brains using the wilcox.test function in R (v4.0.0) with default parameters and the *P*-values were adjusted by Benjamini-Hochberg correction for multiple testing.

**Analysis of candidate *cis*-regulatory elements**

We employed a tiered priority approach to create pseudo-bulk replicates using 'addGroupCoverages' function in ArchR.[69] ATAC peaks were called based on the generated group coverage objects for each cell type. To avoid bias from pseudo-bulk replicates that have very few cells, we provide a cutoff for the upper limit of the number of peaks called per cell type (n = 250,000) and clusters smaller than 100 cells were removed as outliers. We then used

'addReproduciblePeakSet' function from the R package ArchR to add reproducible peak set using MACS2[70] (v2.1.1) in each cell subclusters with respect to different brain regions and disease status. Using those reproducible peak set, we first identified co-accessible peaks where one of the peaks overlaps a gene's promoter, which serves as a candidate target gene for that specific *cis*-regulatory elements. We restricted our search to peaks within a genomic distance of 250 Kb to the TSS of each gene. This co-accessibility analysis was stratified by different cell populations and disease status of each sample. We further performed peak-to-gene linkage analysis using gradient boosting regression approach by leveraging integrated scRNA-seq data to score correlation importance between peak accessibility and gene expression. Spearman rank correlation analysis was used to separate positive (> 0.03) from negative (< -0.03) interactions. We restricted our search to 250 Kb upstream/downstream of the gene body or its promoter region. This procedure was carried out using the 'calculate_regions_to_genes_relationships' function in SCENIC+.[71] We further confirmed these findings by performing a Pearson correlation analysis between the expression of the candidate target gene counts from snRNA-seq with log-normalized accessibility of the candidate linked peak from snATAC-seq. This procedure was carried out using the 'addPeak2GeneLinks' function in ArchR.[69] Links with an absolute correlation score < 0.2 were removed from subsequent analysis.

**Differential expression analysis**

Differentially expressed genes (DEGs) were determined between AD/ADRD and control cerebellum for each cell subclusters. To do so, we first extracted gene expression matrix from ArchR object and then transformed the ArchR object to SummarizedExperiment object using

‘getMatrixFromProject’ function in ArchR according to standard protocols. Within each cell cluster, the gene expression data was log-normalized with gene expression counts. We next used ‘sc.tl.rank_genes_groups’ function in scanpy[72] (v0.1.0) to perform differential expression analysis for genes between AD/ADRD and control cerebellum. Genes with a Benjamini-Hochberg-corrected $P$ value < 0.05 and an absolute $\log_2$FC $\geq$ 1.0 were determined to be biological significance.

**Single-nucleus transcription factor binding motif analysis**

We first used cisTopic to classify cCREs into different regulatory topics and to cluster cells based on their regulatory topic contributions.[73] In total, we identified 45 high accurate regulatory topics based on the snATAC-seq data models (**Figure S11A**). Then we identified highly variable regions to speed up the hypothesis testing step for identifying differentially accessible regions (DARs) (**Figure S11B**). The inferred ‘*cis*-regulatory topics’ were further exploited for differentially accessible regions analysis between AD/ADRD and control cerebellum. Together with regulatory topics, we can also identify differentially accessible regions between AD/ADRD and control cerebellum. We first used a scale factor ($n = 10^{-6}$) to shrink very low probability values and then impute the region accessibility to log-normalized data. Then we performed a Wilcoxon rank-sum test between each group in specified contrast. DARs with an adjusted $P$ value < 0.05 and an absolute $\log_2$FC $\geq$ 0.3 were determined to be biologically significant. The inferred ‘*cis*-regulatory topics’ and DARs can be directly exploited for motif enrichment analysis by pycisTarget suite in SCENIC+ workflow, which includes more than 49,504 motifs from 29 motif collections, with curated TF motif annotations based on direct evidence and ortholog relationships between species for human, mouse, and fly.[71] By default, motif enrichment is run using topics or DARs as foreground and 500 regions in other topics/DARs as background and then a Wilcox test is performed between foreground and background regions sets to assess enrichment. For each motif, we used a set of regions to calculate enrichment score (AUC) and ranking database containing ranked motif scores. Enrichment was calculated as a normalized AUC at the top 0.5% ranking (NES):

$$\mathrm{NES} = \frac{\mathrm{AUC} - \mathrm{mean(AUC)}}{\mathrm{s.d.(AUC)}} \quad (1)$$

Where the mean (AUC) represents the average AUC value across all motifs and s.d.(AUC) represents the standard deviation of AUC values across all motifs.

For enriched TFs of interest, we used chromVAR[74] to compute enrichment of TF activity on a per-cell basis from global chromatin accessibility data. Then we used ArchR to identify a set of background peaks that are matched by GC-content and accessibility. Finally, ArchR uses this background set of peaks and global accessibility to compute bias-corrected deviations with chromVAR for each sample independently. We further used 'getFootprints' function in ArchR to perform TF footprinting analysis in pseudo-bulk aggregates of single nuclei in the same cell type of interest, splitting nuclei based on disease status or cell types.

**Inference of gene regulatory networks**

We used SCENIC+ to build enhancer-related gene regulatory networks (eGRNs) in human cerebellum. Briefly, we first used raw gene expression counts to predict raw TF expression, which can be further used to calculate TF-to-gene importance scores using gradient-boosting machine regression implemented in Arboreto python package (v0.1.6).[71] We retained both positive (> 0.03) and negative (< -0.03) interactions from Pearson correlation analysis. Combining with the identified region-to-gene importance scores, we further binarized those scores into three different categories: 1) taking the 85th, 90th and 95th quantile of the region-to-gene importance scores, 2) taking the top 5, 10 and 15 regions per gene based on the region-to-gene importance scores, and 3) performing a custom implementation of the BASC[75] method on the region-to-gene importance scores. Lastly, we created the eGRNs by taking all regions that are enriched for a TF and all genes that linked to those regions. After ranking all genes based on their TF-to-gene important scores, we used gsea_compute function from GSEApy (v 0.10.8) to perform a gene set enrichment analysis (GSEA) by calculating the enrichment of gene sets within each eGRNs. We considered the genes in the top of the ranking were the targets of eGRNs. Correlations with positive and negative interaction were analyzed separately. We used ctxcore python package to perform enrichment analysis for eGRN target regions and target genes. eGRNs with more than 40 target genes and 200 target regions were selected for subsequent analysis.

**Trajectory analysis for granule cells**

We ordered granule cells in pseudo-time to create a cellular trajectory that approximates the differentiation of granule cell precursors into fully differentiated granule cells. We used the 'addTrajectory' function in ArchR to create the trajectory and added this trajectory to our ArchRProject. We further used scVelo[76] to validate the trajectory of ArchR. scVelo has enabled the recovery of directed dynamic information by leveraging the fact that newly transcribed, unspliced pre-mRNAs and mature, spliced mRNAs can be distinguished in common single-cell RNA-seq protocols.[76] This latent time represents the real time experienced by cells as they differentiated. We overlayed the gene expression and chromatin accessibility data on the trajectory within UMAP embedding. To visualize changes across pseudo-time, we retrieved the trajectory of interest from the ArchRProject using the 'getTrajectory' function. To identify driver TFs or genes of differentiation, we further performed integrative pseudo-time analyses by integrating of gene expression with motif or chromatin accessibility across pseudo-time using 'correlateTrajectories' function in ArchR.[69] We used 'plotTrajectoryHeatmap' function in ArchR to create the heatmap for the trajectory of interest.

**Partitioned heritability analysis using snATAC-seq data**

We used LDSC (v 1.0.1) to estimate heritability of multiple brain disorders in each clustered cell type in our dataset.[77] We first analyzed the partitioned heritability of GWAS summary statistics SNPs conditioned on baseline model LD scores by functional categories, and then jointly modeling with regression weights and allele frequencies. This procedure was performed using make_annot.py script in LDSC. Then we performed a cell type-specific analysis by using cluster-specific peaks as input functional categories for LDSC. Clusters with fewer than 40 cells were removed from subsequent analysis. We generated cluster-specific peaks by overlapping the originally identified peaks from a given cluster with the union peak set from that specific cluster. Publicly available GWAS statistics data were collected for AD,[10,42,43,78,79] LBD,[80] ALS,[81,82] PD,[46,83] cerebellar volume,[84] attention-deficit/hyperactivity disorder (ADHD),[85] depression,[86] insomnia,[87] and neuroticism.[88] Detailed information of GWAS data used in this study can be found in **Table S11**. LD scores were computed for each set using ldsc.py script. Cell type-specific partitioned heritability analysis was performed according to recommend pipelines using ldsc.py script with parameter --ref-ld-chr-cts. Benjamini-Hochberg multiple-testing approach was applied to adjust the heritability enrichment *P* values.

**Analysis of fine-mapped GWAS variants**

We used three approaches to identified fine-mapped GWAS SNPs. We first selected SNPs passing genome-wide significance (GWAS $P < 5.0 \times 10^{-8}$) in the GWAS summary statistics datasets we mentioned above. Then we selected SNPs from genome wide significant GWAS loci that co-localized with expression quantitative trait loci from human cerebellum using coloc[89] (v 5.1.0) and FINEMAP[90] approaches. SNPs with a posterior probability >0.01 were selected for subsequent analysis. Finally, we added any SNPs in linkage disequilibrium (LD) with each SNPs identified in the previous two categories defined as a LD $R^2 > 0.8$ calculated from phase 3 genotypes of individuals of European ancestry in the 1000 Genomes dataset. In total, we identified 3,884 unique SNPs across 196 loci in AD, 4,579 unique SNPs across 160 loci in PD, 656 unique SNPs across 144 loci in ALS, 141 unique SNPs across 29 loci in LBD and 662 unique SNPs across 65 loci in cerebellar volume. A list of all fine-mapped GWAS loci used in this study is provided in **Table S12**.

**Statistics**

All statistical methods and tests used in this paper are described in the figure legends, Methods, Supplementary files, or main text as appropriate.

### Data and code availability

Raw snMultiome sequencing data generated from this study have been deposited in the Gene Expression Omnibus (GEO) database under the accession number GSE##### and the AD Knowledge Portal (SynapseID, https://adknowledgeportal.synapse.org/ [Access ID will be available upon publication]). Codes used for snMultiome analysis are available in the GitHub repository: https://github.com/ChengF-Lab/snMulti-Ome. All other data are provided in Supplementary Tables 1-13.

### Acknowledgements

**Funding:** This work was primarily supported by the National Institute on Aging (NIA) under Award Number U01AG073323, R21AG083003, R01AG066707, R01AG076448, R01AG082118, R01AG084250, and RF1AG082211, and the National Institute of Neurological Disorders and Stroke (NINDS) under Award Number RF1NS133812, and Alzheimer's Association (ALZDISCOVERY-1051936) to F.C. This work was supported in part by NIA grants 3R01AG066707-01S1, 3R01AG066707-02S1, and R56AG074001 to F.C. This work was supported in part by the Translational Therapeutics Core of the Cleveland Alzheimer's Disease Research Center (NIH/NIA: P30AG072959) to F.C., A.A.P., J.B.L. and J.C. This work was supported in part by NIH/NINDS U54NS100717 and NIA R01AG054214 to L.G. This work was supported in part by the Brockman Foundation, Project 19PABH134580006-AHA/Allen Initiative in Brain Health and Cognitive Impairment, the Elizabeth Ring Mather & William Gwinn Mather Fund, S. Livingston Samuel Mather Trust, and the Louis Stokes VA Medical Center resources and facilities to A.A.P. This work was supported in part by Keep Memory Alive (KMA), NIGMS grant P20GM109025, NINDS grant U01NS093334, NIA grant R01AG053798 and R35AG071476, and the Alzheimer’s Disease Drug Discovery Foundation (ADDF) to J.C.

### Author contributions

F.C. conceived the study. Y.F. performed all genetic and genomic data analyses and experiments. M.E.F., B.B., P.J., R.J.C., and Q.M. lead the brain sample collection and preparation. X.C., H.G., and Y.L. help sequencing data generation. J.X., Y.H., W.M., P.T.N., J.B.L., A.A.P., and J.C. interpreted the data analysis. Y.F., M.E.F., H.G. and F.C. drafted the manuscript. Y.F., F.C., M.E.F., A.A.P. and J.C. critically revised the manuscript. All authors gave final approval of the manuscript.

### Competing interests

Dr. Cummings has provided consultation to AB Science, Acadia, Alkahest, AlphaCognition, ALZPathFinder, Annovis, AriBio, Artery, Avanir, Biogen, Biosplice, Cassava, Cerevel, Clinilabs, Cortexyme, Diadem, EIP Pharma, Eisai, GatehouseBio, GemVax, Genentech, Green Valley, Grifols, Janssen, Karuna, Lexeo, Lilly, Lundbeck, LSP, Merck, NervGen, Novo Nordisk, Oligomerix, Ono, Otsuka, PharmacotrophiX, PRODEO, Prothena, ReMYND, Renew, Resverlogix, Roche, Signant Health, Suven, Unlearn AI, Vaxxinity, VigilNeuro pharmaceutical, assessment, and investment companies. Dr. Leverenz has received consulting fees from consulting fees from Vaxxinity, grant support from GE Healthcare and serves on a Data Safety Monitoring Board for Eisai. The other authors have declared no competing interests.

### Figure Legends

**Figure 1 Cellular diversity in the diseased brain revealed by single nuclei multiomics.** (**A**) Schematic of the samples and sequencing experiments used in this study, created with BioRender.com. (**B**) Immunostaining of amyloid-beta using 4G8 antibody in DLBD cerebellum and dentate nucleus using AT8 antibody in PSP cerebellum. (**C**) Uniform manifold approximation and projection (UMAP) visualization of the 103,861 brain nucleus profiled with snATAC-seq (left), snRNA-seq (middle), and jointly snATAC-seq and snRNA-seq (right), colored by the annotated clusters. OLs, oligodendrocytes; Gran, granule cells; Astro, astrocytes; Micro, microglia; Glu, glutamate neurons; Excit, excitatory neuron; OPCs, oligodendrocyte progenitor cell. (**D**) Raw-normalized gene expression of selected marker genes for each snRNA cluster. Color indicates scaled mean expression across all clusters and dot size indicates fraction of expressing cells in that cluster. (**E**) Raw-normalized gene activity score of selected marker genes shown in **D** for each snRNA cluster. (**F**) Proportion of cells from each sample comprising each cluster in the context of brain region. Measures of cellular composition changes for each cluster between diseased and control cerebellum were analyzed using Wilcoxon test. * $P < 0.05$.

**Figure 2 Identification of candidate *cis*-regulatory elements.** (**A**) Tn5 bias-subtracted TF footprinting analysis for *RFX3* and *ATF1* by cell clusters of snATAC. The upper panel shown TF binding motif logo. (**B**) Schematic of peak-to-gene linkages analysis using the full snATAC and snRNA datasets and the five granule cells subclustered datasets. Linkages were analyzed separately and then merged to generate the full set of peak-to-gene linkage sets. (**C**) Heatmap of raw-normalized chromatin accessibility and gene expression for the 431,834 peak-to-gene linkages, which were clustered based on *k*-means clustering analysis. Genes highlighted were well-known GWAS genes from GWAS catalog and key transcription factors involved in granule cell differentiation. (**D**) Genes ranked by the number of significant peak-to-gene associations identified for each gene. The inflection point was set to 150 peak-to-gene linkages and 1,821 genes had >150 peak-to-gene linkages. (**E**) Genomic tracks for chromatin accessibility around 250 Kb flanking regions of *BIN1* locus in cerebellum (top) and frontal cortex (bottom). (**F**) Genomic tracks for chromatin accessibility around 250 Kb flanking regions of *IL33* locus in cerebellum (top) and frontal cortex (bottom). Peak-to-gene linkages were shown as loops below those genomic tracks and colored by linked correlation value.

**Figure 3 Cell type-specific transcriptomic changes in diseased cerebellum.** (**A**) Raw-normalized $\log_2$FC of all up- and downregulated genes in diseased cerebellum. (**B**) Upset plot showing the size of overlaps between the sets of up- (top) and downregulated (bottom) genes identified in each cell type. Bar plot on the top shows the number of overlapping genes between multiple cell types or a unique cell type. (**C**) Venn diagrams showing overlaps between cCREs-linked genes, genes differentially expressed in a specific cell type (cell-type DEGs) and genes differentially expressed in diseased cerebellum (diagnosis DEGs). One-sided Fisher's exact test was used for gene-set overlap significance ($P < 0.05$). (**D**) Two-sided bar plot showing number of up- (right) and downregulated (left) genes for each cell type in the cerebellum of disease context, including AD/ADRD, AD, DLBD and PSP. (**E**) Dot plot showing log-transformed enrichR combined scores for GO terms for differentially expressed gene sets in the cerebellum of disease context, including AD/ADRD, AD, DLBD and PSP. Upset plot on the right showing the size of overlaps between different disease context identified in each enriched term. (**F**) Genomic tracks for chromatin accessibility around the *CALM1* (left), *TMEM160* (middle) and *ARHGDIG* (right) locus in AD/ADRD cerebellum. Violin plot on the right showing expression level of gene under consideration for specific cell type. Peak-to-gene linkages were shown as loops below those genomic tracks and colored by linked correlation value.

**Figure 4 Identification of enhancer-associated gene-regulatory networks in diseased cerebellum.** (**A**) Schematic of defining peak-gene-TF trios. The accessibility of a linked peak harboring a specific TF motif must be correlated with the mRNA level of that TF and the expression of that TF must be correlated with the linked gene for that peak. (**B**) Heatmap/dot-plot showing TF expression of the top eGRNs. Color indicates normalized TF expression and dot size indicates cell-type specificity (RSS) score. (**C**) Left: snMultiome UMAP colored by *RORA* motif variability (top) and its target gene score (bottom). Right: Tn5 bias-subtracted TF footprinting analysis for *RORA* by snATAC granule and Purkinje cell clusters (top) and by disease status (bottom). TF binding motif shown as motif logo above. (**D**) Left: snMultiome UMAP colored by *ELF1* motif variability (top) and its target gene score (bottom). Right: Tn5 bias-subtracted TF footprinting analysis for *ELF1* by snATAC granule and Purkinje cell clusters (top) and by disease status (bottom). TF binding motif shown as motif logo above. (**E**) Visualization of *RORA*-gene regulatory networks in AD/ADRD Purkinje cells. (**F**) Visualization of TF-gene regulatory networks formed by *ELF1* and *CHD2* in AD/ADRD mature granule cells.

**Figure 5 snMultiome granule cell trajectory analyses.** (**A**) Differentiation trajectory starting from granule cell progenitor to mature granule cells using snMultiome data. (**B**) RNA velocity revealed differentiation trajectory starting from granule cell progenitor to mature granule cells using snRNA data. (**C**) Dot plot showing gene expression of *TCF12* and *RORA* colored by pseudo-time. (**D**) Paired heatmap showing gene regulators whose chromatin accessibility (left) and matched gene expression (right) are positively correlated across granule cell pseudo-time trajectory. (**E** and **G**) Dot plot showing gene expression of *PDE4B* and *RNF152* colored by pseudo-time. (**F** and **H**) Genomic tracks for chromatin accessibility around the *PDE4B* (**F**) and *RNF152* (**H**) locus in granule cells of AD/ADRD cerebellum. Violin plot on the right showing expression level of gene under consideration. Peak-to-gene linkages were shown as loops below those genomic tracks and colored by linked correlation value.

**Figure 6 Identification of cell types and genes associated with disease risk loci.** (**A**) Heatmap showing Linkage Disequilibrium Score Regression (LDSC) enrichment score for various neurodegenerative conditions in peak regions of snATAC clusters. FDR-corrected *P* values are overlaid on the heatmap (*$q < 0.05$ and **$q < 0.005$). (**B**) One-sided Fisher's exact test enrichment of fine-mapped, disease-related GWAS SNPs in cell type-specific differentially accessible peaks in diseased cerebellum. Color and dot size indicate FDR-corrected $-\log_{10}P$ value. GWAS traits are grouped as in **A**. (**C**) Identification of likely causal GWAS SNPs and linked genes in diseased cerebellum. Left: Manhattan plot showing the $-\log_{10}(P$ value) distribution of GWAS loci across different neurodegenerative conditions. Diseased-associated SNPs identified by colocalization analysis and fine-mapping are colored by green. All the SNPs shown in this study are annotated with assembly GRCh38. Middle: heatmap showing raw-normalized $\log_2$FC of GWAS-linked genes in AD/ADRD, AD and ADRD cerebellum. Right:

barplot showing number of linked peaks, number of linked causal SNPs and the mean of fine-mapped posterior probability for linked causal SNPs per gene.

**Figure 7 Linking causal variants to target genes through Hi-C chromatin looping.** (**A**) Normalized chromatin accessibility landscape for cell type-specific pseudobulk tracks around the *SEZ6L2* (Seizure 6-like protein 2) locus. Top: Interaction maps between promoter of *SEZ6L2* and differentially accessible peak containing GWAS SNP rs4788201. Middle: Genomic tracks for chromatin accessibility around the *SEZ6L2* locus in AD/ADRD cerebellum. Violin plot on the right showing expression level of *SEZ6L2* for all cell clusters in cerebellum. Peak-to-gene linkages were shown as loops below those genomic tracks and colored by linked correlation value. Bottom: LocusCompare plots for high-probability genome-wide colocalized loci. The colocalized SNPs are labeled with variant identifiers and annotated as diamonds. Plots are colored based on linkage disequilibrium (LD) bins relative to the lead SNPs (red, ≥ 0.8; orange, 0.6-0.8; green, 0.4-0.6; light blue, 0.2-0.4; and dark blue, < 0.2). The SNP pairwise LD data were calculated based on the 1000 Genomes Phase 3 (ALL) reference panel. (**B**) Normalized chromatin accessibility landscape for cell type-specific pseudobulk tracks around *KANSL1* (KAT8 Regulatory NSL Complex Subunit 1) locus.

### Supplementary Figure Legends

**Figure S1. Single-nucleus multiome quality control matrix, Related to Figure 1**. (**A**, **B**) Violin plots depicting the TSS enrichment score per nucleus (**A**) and number of detected ATAC fragments per nucleus (**B**). (**C**, **D**) Ridge plot for each sample for the TSS enrichment scores (**C**) and number of detected ATAC fragments per nucleus (**D**). (**E**) snATAC-seq fragment size distributions of all samples and TSS enrichment profiles. (**F**) Joint UMAP colored by sample batch.

**Figure S2. Selected cell type-specific marker genes, Related to Figure 1.** (**A**, **B**) Heatmap depicting gene activity score (**A**) and gene expression (**B**) of selected cell type-specific marker genes. (**C**) Joint UMAPs for selected marker genes colored by normalized gene expression and gene activity scores.

**Figure S3. Clustering of scMultiome data robustness to subsampling analysis, Related to Figure 1.** (**A**) Repeated dimensionality reduction and clustering of the scMultiome datasets with eight samples (AD_FC4, AD_FC6, AD_CBE1, PSP1, DLBD1, Control1, Control5 and Control8) removed from the full dataset. UMAP representations of the full subsampled dataset using scATAC-seq, scRNA-seq and the scMultiome data. (**B**) Repeated dimensionality reduction and clustering of the scMultiome datasets with 25% of the cells randomly removed from the full dataset. UMAP representations of the full subsampled dataset using scATAC-seq, scRNA-seq and the scMultiome data. We used the same marker genes with **Figures 1D** and **1E** to identify cell subtypes.

**Figure S4. Cellular proportion of nucleus mapping to each cluster of each sample, split by brain regions and disease context, Related to Figure 1.** (**A**) UMAP visualization where dots correspond to individual nuclei for snATAC-seq, snRNA-seq, and joint snATAC-seq and snRNA-seq, colored by brain regions. (**B**) UMAP visualization where dots correspond to individual nuclei for snATAC-seq, snRNA-seq, and joint snATAC-seq and snRNA-seq, colored by disease context. (**C**) Box plots showing the proportion of nucleus mapping to each cluster of each sample, split by disease context. Measures of significance were calculated using Kruskal-wallis rank sum test in R.

**Figure S5. Chromatin accessibility profiles from snMultiome profiles of human cerebellum and frontal cortex reveal cell type-specific epigenetic landscapes, Related to Figure 2.** (**A**) Number of chromatin accessibility peaks for each cell subtypes identified using snATAC-seq data. Peaks were required to be present in at least two pseudo-bulk ATAC replicates. (**B**) UMAPs of motif deviation scores for selected TFs enriched in granule cells (*RFX3*) and Purkinje cells (*ATF1*). (**C**) The candidate *cis*-regulatory elements enriched binding motifs of key transcription factors.

**Figure S6. Identification of candidate *cis*-regulatory elements, Related to Figure 2.** Genomic tracks for chromatin accessibility around 250 Kb flanking regions of *APOE* locus (**A**), *TGFB2* (**B**), *PICALM* (**C**) and *APP* (**D**). Peak-to-gene linkages were shown as loops below those genomic tracks and colored by linked correlation value.

**Figure S7.** Pie charts showing percentage of candidate *cis*-regulatory elements in different functional genomic elements, including promoters, exons, introns, upstream 5 kb region of transcriptional start sites, and downstream 5 kb region of transcriptional start sites, Related to Figure 2.

**Figure S8.** Pseudo-time heatmaps showing motif deviations (A), gene expression matrix (B) and gene score matrix (C) along the trajectory from from granule cell progenitor to mature granule cells, Related to Figure 5.

**Figure S9. Published Hi-C data confirmed the regulatory relationship between candidate cis-regulatory elements and the promoters of 9 genes in human cerebellum, Related to Figure 7.** Target genes are highlighted in each heatmap.

**Figure S10. Analysis of snATAC-seq datasets from publicly available human aging cerebellum, Related to Figure 1.** (**A**) snATAC-seq fragment size distributions and TSS enrichment profiles per sample. (**B**) Violin plots depicting the TSS enrichment score per nucleus and number of detected ATAC fragments per nucleus. (**C**) Joint UMAPs for selected marker genes colored by normalized gene activity scores. (**D**) Joint UMAP colored by sample batch. (**E**) UMAP visualization of aging brain nucleus colored by the annotated clusters. (**F**) Number of chromatin accessibility peaks for each annotated clusters as determined by snATAC-seq. Peaks were identified using ArchR.

**Figure S11. Analysis of enhancer candidate identification and topic modeling using pycisTopic, Related to Figure 4.** (**A**) Model selection of the optimal number of topics. In this case, the optimal number of topics is 45. (**B**) Volcano plot showing highly variable regions among AD/ADRD cases and controls.

## Figures

### Figure 1

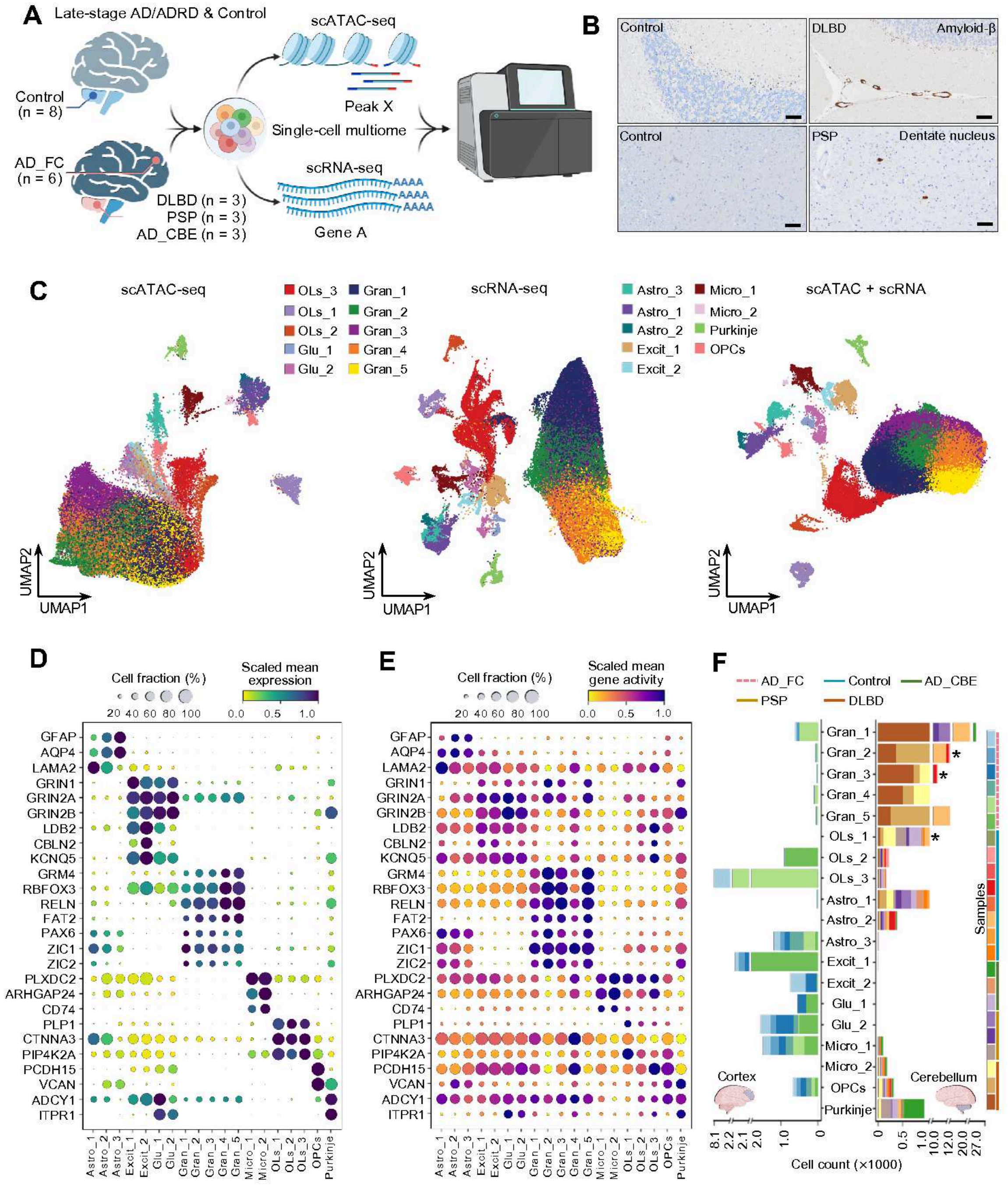

Figure 1

Cellular diversity in the diseased brain revealed by single nuclei multiomics. (A) Schematic of the samples and sequencing experiments used in this study, created with BioRender.com. (B) Immunostaining of amyloid-beta using 4G8 antibody in DLBD cerebellum and dentate nucleus using AT8

antibody in PSP cerebellum. (C) Uniform manifold approximation and projection (UMAP) visualization of the 103,861 brain nucleus profiled with snATAC-seq (left), snRNA-seq (middle), and jointly snATAC-seq and snRNA-seq (right), colored by the annotated clusters. OLs, oligodendrocytes; Gran, granule cells; Astro, astrocytes; Micro, microglia; Glu, glutamate neurons; Excit, excitatory neuron; OPCs, oligodendrocyte progenitor cell. (D) Raw-normalized gene expression of selected marker genes for each snRNA cluster. Color indicates scaled mean expression across all clusters and dot size indicates fraction of expressing cells in that cluster. (E) Raw-normalized gene activity score of selected marker genes shown in D for each snRNA cluster. (F) Proportion of cells from each sample comprising each cluster in the context of brain region. Measures of cellular composition changes for each cluster between diseased and control cerebellum were analyzed using Wilcoxon test. * $P < 0.05$.

## Figure 2

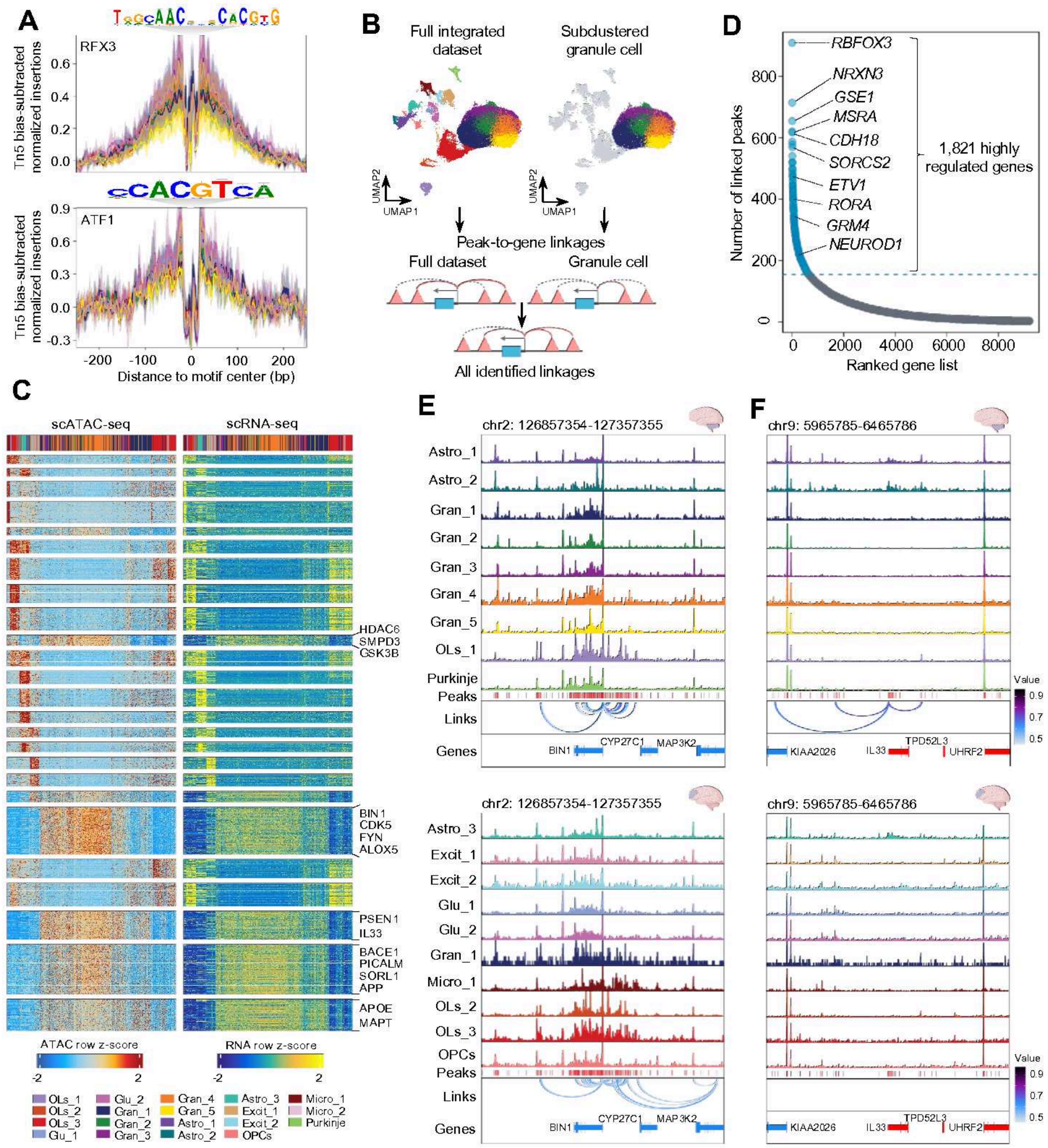

### Figure 2

Identification of candidate cis-regulatory elements. (A) Tn5 bias-subtracted TF footprinting analysis for RFX3 and ATF1 by cell clusters of snATAC. The upper panel shown TF binding motif logo. (B) Schematic of peak-to-gene linkages analysis using the full snATAC and snRNA datasets and the five granule cells subclustered datasets. Linkages were analyzed separately and then merged to generate the full set of peak-to-gene linkage sets. (C) Heatmap of raw-normalized chromatin accessibility and gene expression for the 431,834 peak-to-gene linkages, which were clustered based on k-means clustering analysis. Genes highlighted were well-known GWAS genes from GWAS catalog and key transcription factors involved in granule cell differentiation. (D) Genes ranked by the number of significant peak-to-gene associations identified for each gene. The inflection point was set to 150 peak-to-gene linkages and 1,821 genes had >150 peak-to-gene linkages. (E) Genomic tracks for chromatin accessibility around 250 Kb flanking regions of BIN1 locus in cerebellum (top) and frontal cortex (bottom). (F) Genomic tracks for chromatin accessibility around 250 Kb flanking regions of IL33 locus in cerebellum (top) and frontal cortex (bottom). Peak-to-gene linkages were shown as loops below those genomic tracks and colored by linked correlation value.

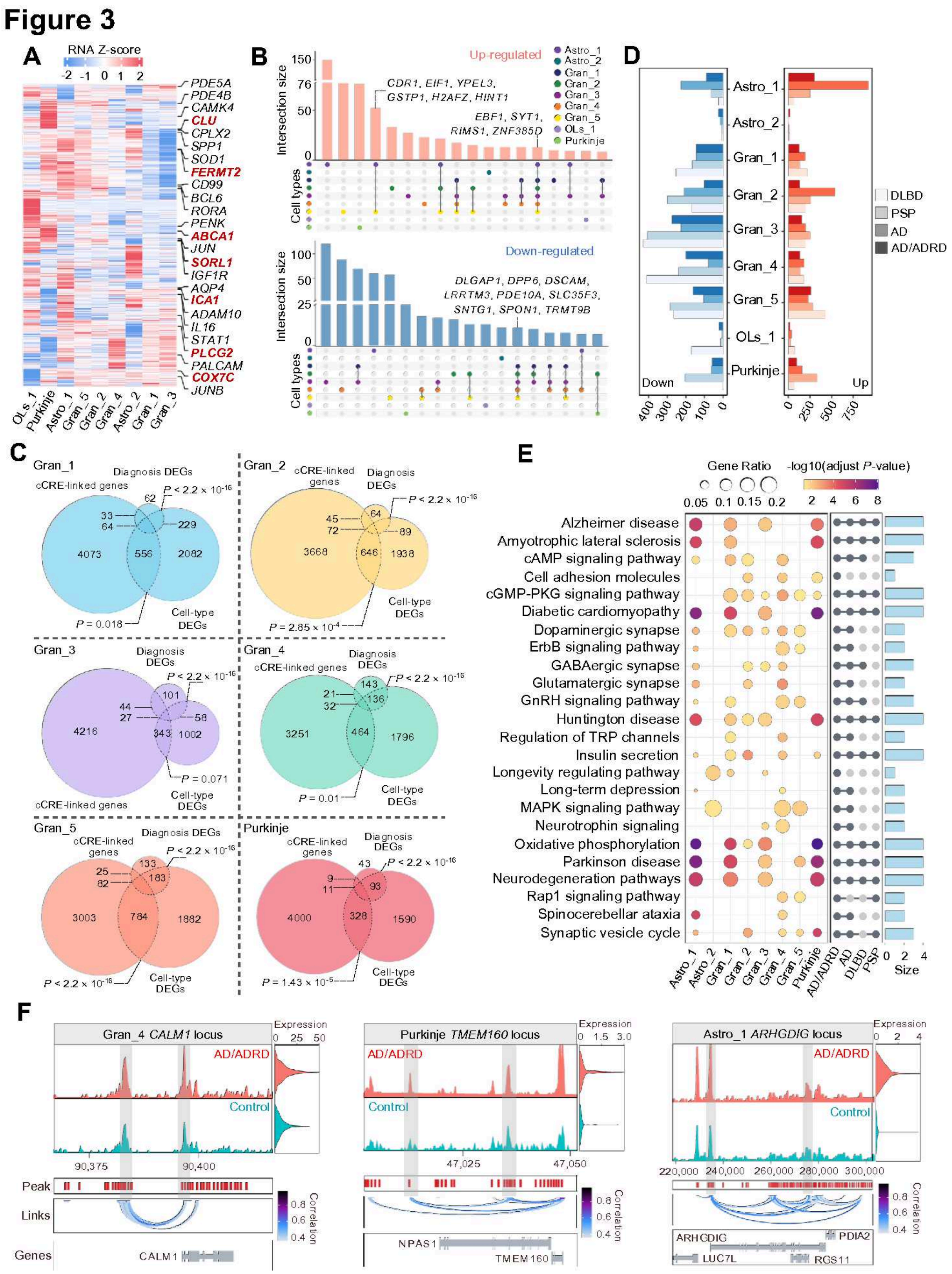

### Figure 3

Cell type-specific transcriptomic changes in diseased cerebellum. (A) Raw normalized log2FC of all up- and downregulated genes in diseased cerebellum. (B) Upset plot showing the size of overlaps between the sets of up- (top) and downregulated (bottom) genes identified in each cell type. Bar plot on the top shows the number of overlapping genes between multiple cell types or a unique cell type. (C) Venn diagrams showing overlaps between cCREs-linked genes, genes differentially expressed in a specific cell type (cell-type DEGs) and genes differentially expressed in diseased cerebellum (diagnosis DEGs). One sided Fisher's exact test was used for gene-set overlap significance ($P < 0.05$). (D) Two-sided bar plot showing number of up- (right) and downregulated (left) genes for each cell type in the cerebellum of disease context, including AD/ADRD, AD, DLBD and PSP. (E) Dot plot showing log-transformed enrichR combined scores for GO terms for differentially expressed gene sets in the cerebellum of disease context, including AD/ADRD, AD, DLBD and PSP. Upset plot on the right showing the size of overlaps between different disease context identified in each enriched term. (F) Genomic tracks for chromatin accessibility around 1192 the CALM1 (left), TMEM160 (middle) and ARHGDIG (right) locus in AD/ADRD cerebellum. Violin plot on the right showing expression level of gene under consideration for specific cell type. Peak-to-gene linkages were shown as loops below those genomic tracks and colored by linked correlation value.

## Figure 4

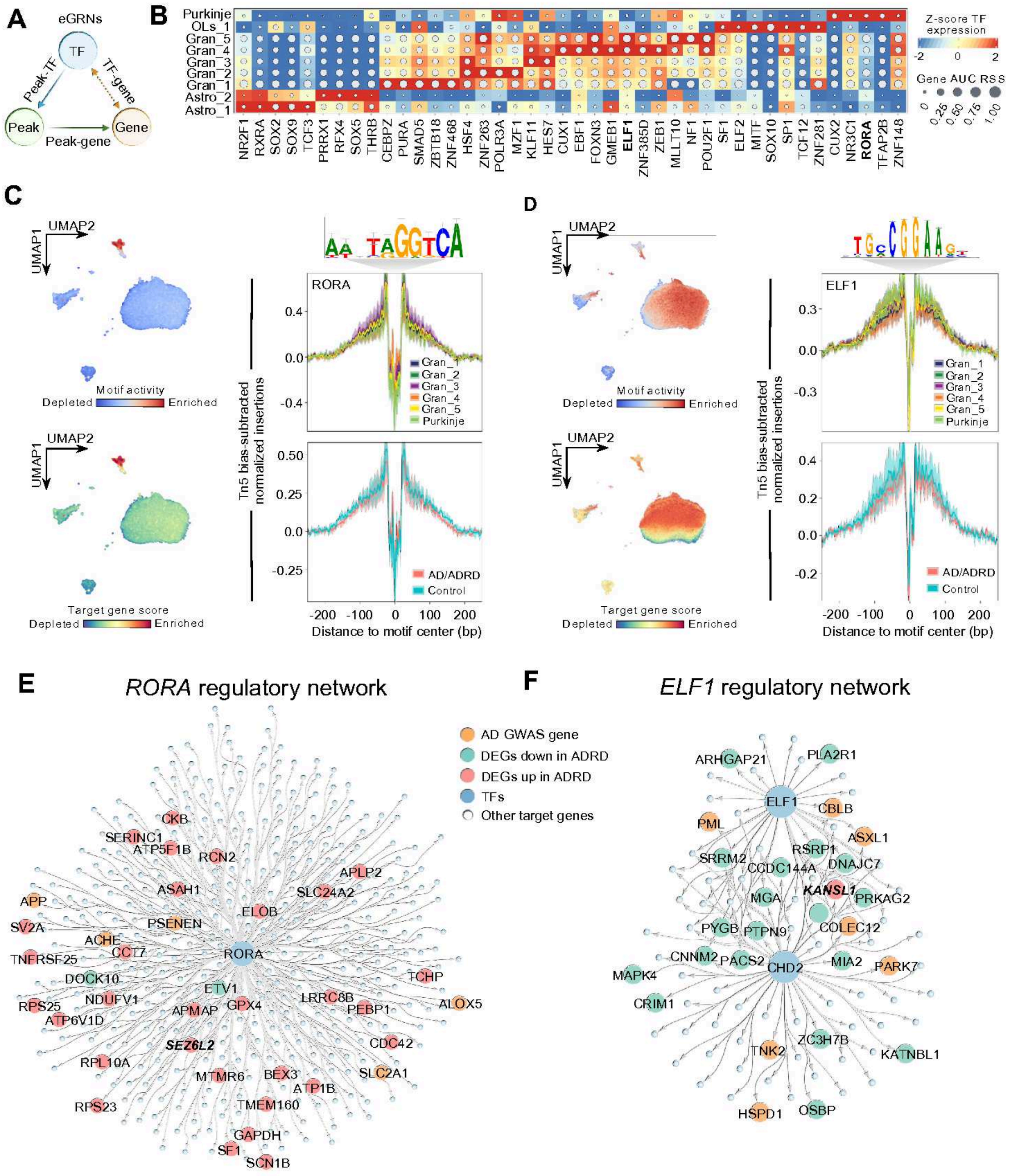

### Figure 4

Identification of enhancer-associated gene-regulatory networks in diseased cerebellum. (A) Schematic of defining peak-gene-TF trios. The accessibility of a linked peak harboring a specific TF motif must be correlated with the mRNA level of that TF and the expression of that TF must be correlated with the linked gene for that peak. (B) Heatmap/dot plot showing TF expression of the top eGRNs. Color indicates normalized TF expression and dot size indicates cell-type specificity (RSS) score. (C) Left: snMultiome

UMAP colored by RORA motif variability (top) and its target gene score (bottom). Right: Tn5 bias-subtracted TF footprinting analysis for RORA by snATAC granule and Purkinje cell clusters (top) and by disease status (bottom). TF binding motif shown as motif logo above. (D) Left: snMultiome UMAP colored by ELF1 motif variability (top) and its target gene score (bottom). Right: Tn5 bias-subtracted TF footprinting analysis for ELF1 by snATAC granule and Purkinje cell clusters (top) and by disease status (bottom). TF binding motif shown as motif logo above. (E) Visualization of RORA-gene regulatory networks in AD/ADRD Purkinje cells. (F) Visualization of TF-gene regulatory networks formed by ELF1 and CHD2 in AD/ADRD mature granule cells.

Figure 5

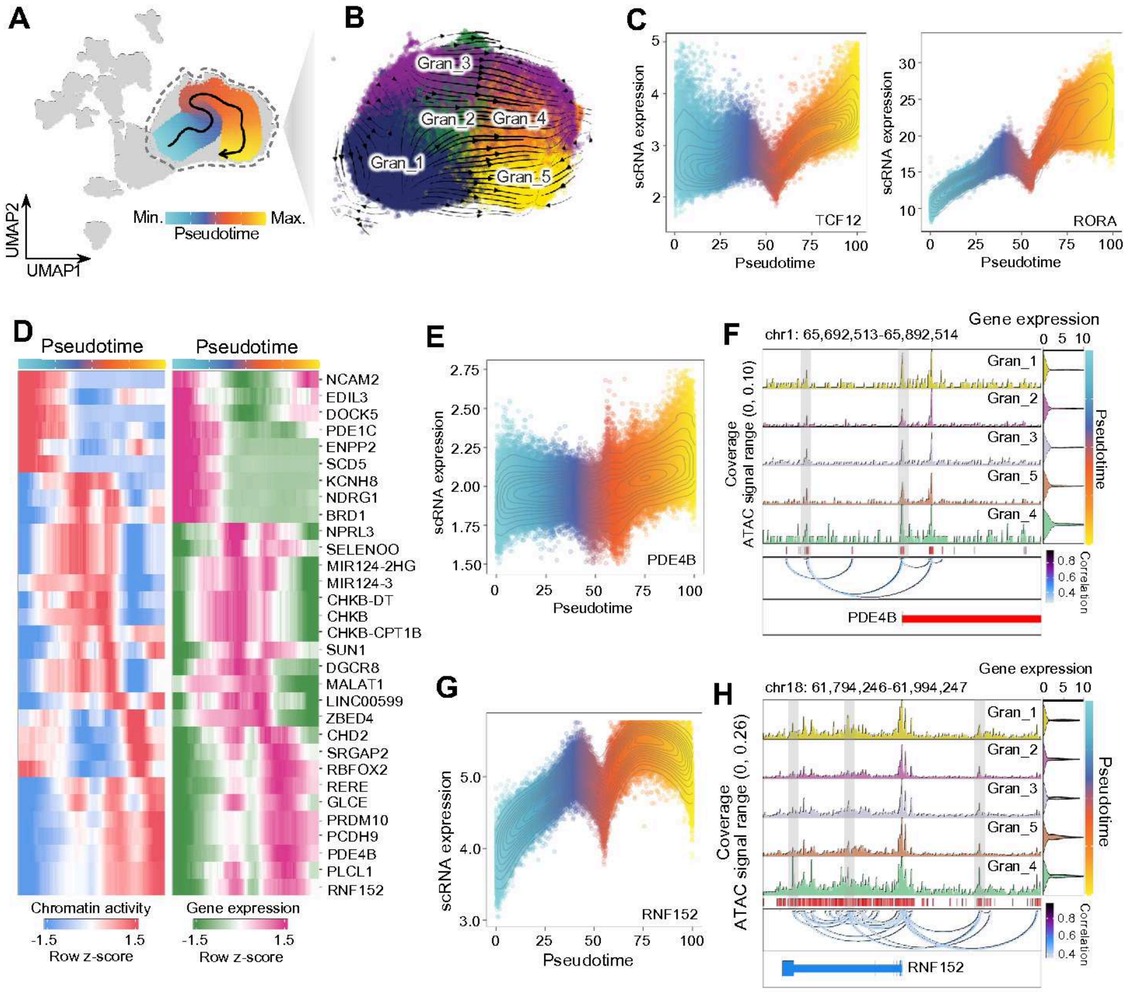

### Figure 5

snMultiome granule cell trajectory analyses. (A) Differentiation trajectory starting from granule cell progenitor to mature granule cells using snMultiome data. (B) RNA velocity revealed differentiation trajectory starting from granule cell progenitor to mature granule cells using snRNA data. (C) Dot plot showing gene expression of TCF12 and RORA colored by pseudo-time. (D) Paired heatmap showing gene regulators whose chromatin accessibility (left) and matched gene expression (right) are positively correlated across granule cell pseudo-time trajectory. (E and G) Dot plot showing gene expression of PDE4B and RNF152 colored by pseudo-time. (F and H) Genomic tracks for chromatin accessibility around the PDE4B (F) and RNF152 (H) locus in granule cells of AD/ADRD cerebellum. Violin plot on the right showing expression level of gene under consideration. Peak-to-gene linkages were shown as loops below those genomic tracks and colored by linked correlation value.

**Figure 6**

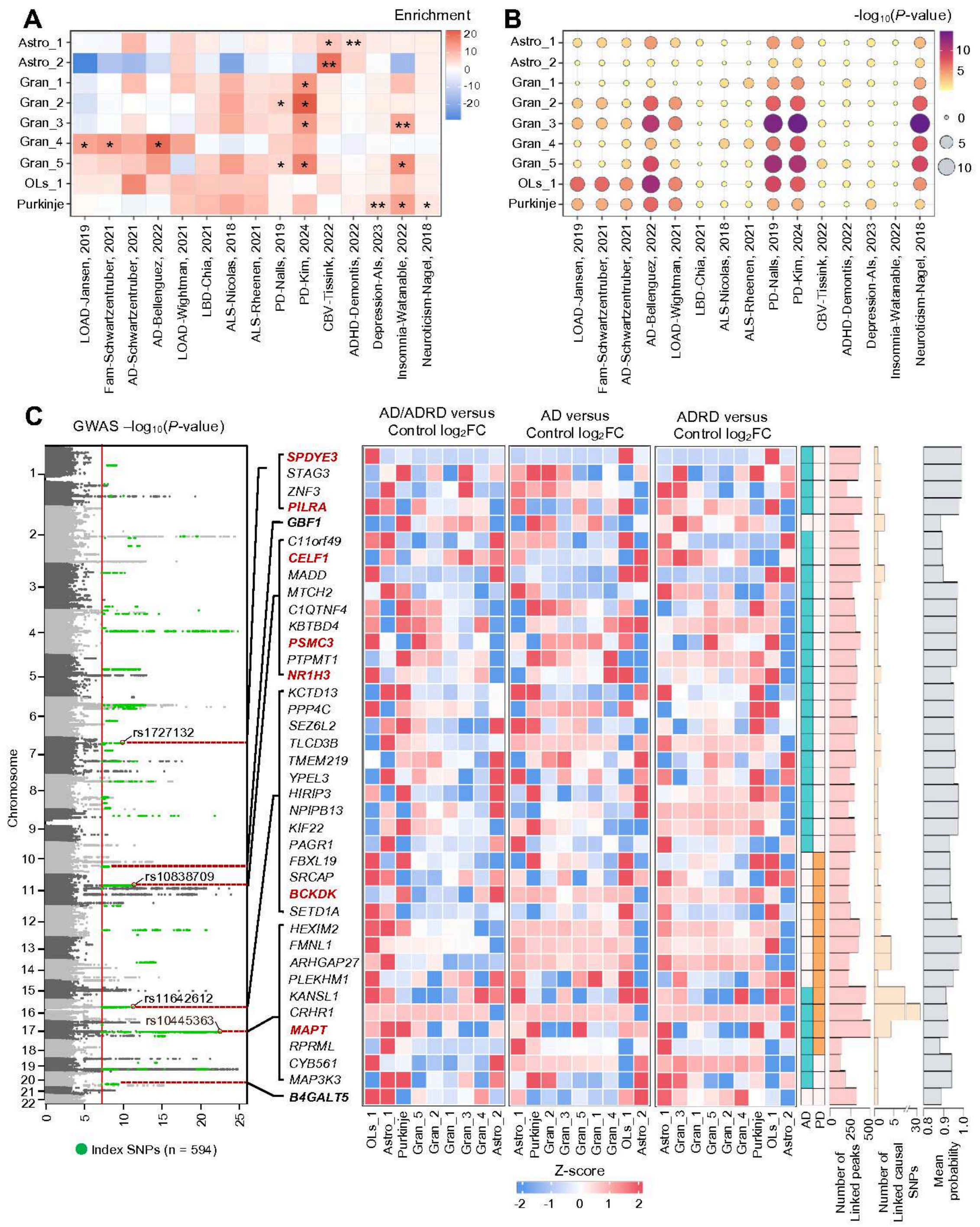

Figure 6

Identification of cell types and genes associated with disease risk loci. (A) Heatmap showing Linkage Disequilibrium Score Regression (LDSC) enrichment score for various neurodegenerative conditions in peak regions of snATAC clusters. FDR-corrected P values are overlaid on the heatmap (*q < 0.05 and **q < 0.005). (B) One-sided Fisher's exact test enrichment of fine-mapped, disease-related GWAS SNPs in cell type-specific differentially accessible peaks in diseased cerebellum. Color and dot size indicate FDR-corrected -log10P value. GWAS traits are grouped as in A. (C) Identification of likely causal GWAS SNPs and linked genes in diseased cerebellum. Left: Manhattan plot showing the -log10(P value) distribution of GWAS loci across different neurodegenerative conditions. Diseased-associated SNPs identified by colocalization analysis and fine-mapping are colored by green. All the SNPs shown in this study are annotated with assembly GRCh38. Middle: heatmap showing raw normalized log2FC of GWAS-linked genes in AD/ADRD, AD and ADRD cerebellum. Right: barplot showing number of linked peaks, number of linked causal SNPs and the mean of fine mapped posterior probability for linked causal SNPs per gene.

Figure 7

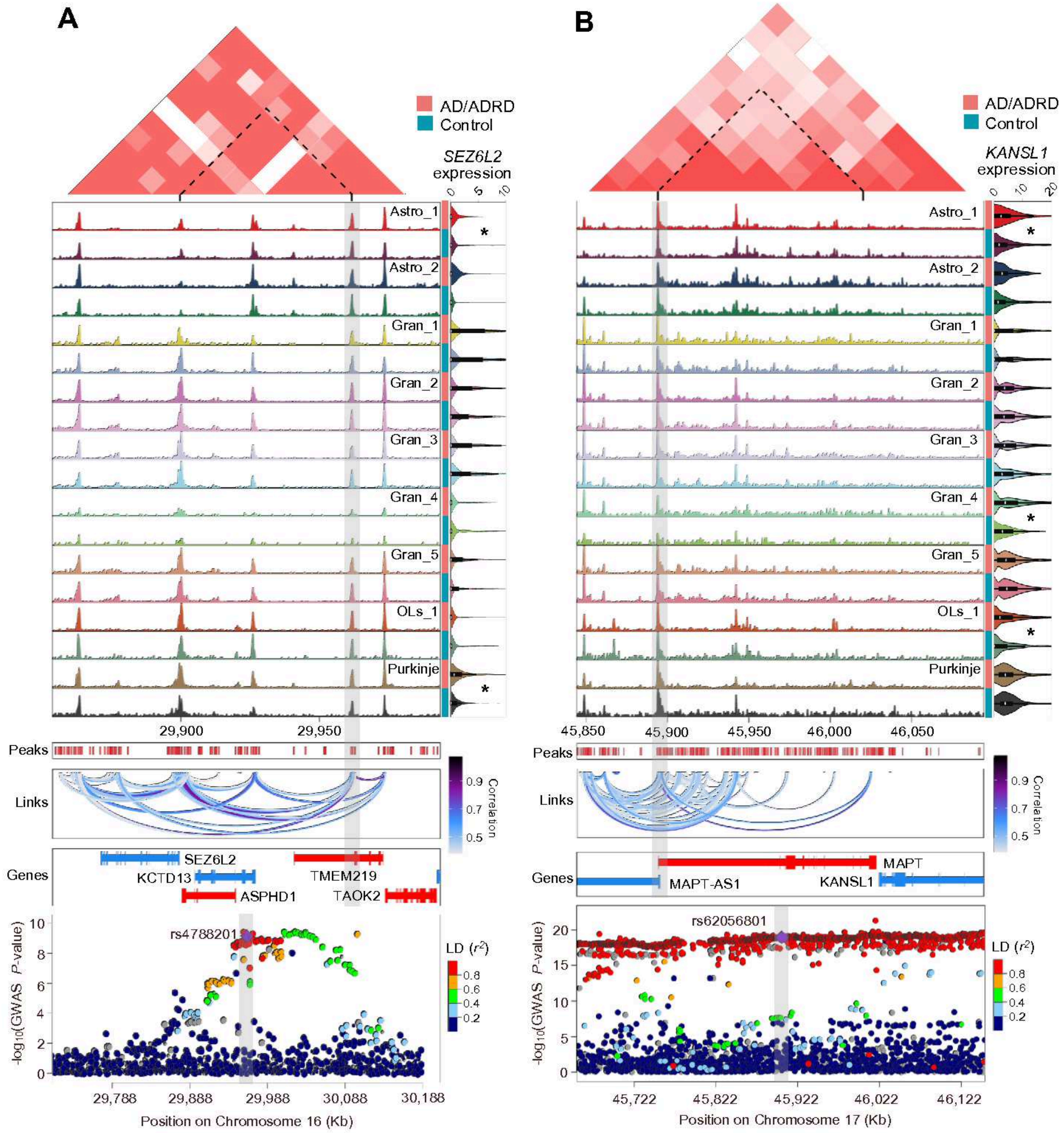

### Figure 7

Linking causal variants to target genes through Hi-C chromatin looping. (A) Normalized chromatin accessibility landscape for cell type-specific pseudobulk tracks around the SEZ6L2 (Seizure 6-like protein 2) locus. Top: Interaction maps between promoter of SEZ6L2 and differentially accessible peak containing GWAS SNP rs4788201. Middle: Genomic tracks for chromatin accessibility around the SEZ6L2 locus in AD/ADRD cerebellum. Violin plot on the right showing expression level of SEZ6L2 for all cell clusters in cerebellum. Peak-to-gene linkages were shown as loops below those genomic tracks and colored by linked correlation value. Bottom: LocusCompare plots for high-probability genome-wide colocalized loci. The colocalized SNPs are labeled with variant identifiers and annotated as diamonds. Plots are colored based on linkage disequilibrium (LD) bins relative to the lead SNPs (red, ≥ 0.8; orange, 0.6-0.8; green, 0.4-0.6; light blue, 0.2-0.4; and dark blue, < 0.2). The SNP pairwise LD data were calculated based on the 1000 Genomes Phase 3 (ALL) reference panel. (B) Normalized chromatin accessibility landscape for cell type-specific pseudobulk tracks around KANSL1 (KAT8 Regulatory NSL Complex Subunit 1) locus.

### Supplementary Files

This is a list of supplementary files associated with this preprint. Click to download.

- SupplementaryFigureS1S11Cheng.pdf
